# Quantification of Giant Unilamellar Vesicle Fusion Products by High-Throughput Image Analysis

**DOI:** 10.3390/ijms24098241

**Published:** 2023-05-04

**Authors:** Adriano Caliari, Martin M. Hanczyc, Masayuki Imai, Jian Xu, Tetsuya Yomo

**Affiliations:** 1 Laboratory of Biology and Information Science, School of Life Sciences, East China Normal University, Shanghai 200062, China; 2 Laboratory for Artificial Biology, Department of Cellular, Computational and Integrative Biology (CIBIO), University of Trento, Polo Scientifico e Tecnologico Fabio Ferrari, Polo B, Via Sommarive 9, 38123 Povo, Italy; 3 Department of Physics, Graduate School of Science, Tohoku University, 6-3 Aramaki, Aoba, Sendai 980-8578, Japan

**Keywords:** giant unilamellar vesicles, vesicle fusion, fluorescence image analysis, high-throughput

## Abstract

Artificial cells are based on dynamic compartmentalized systems. Thus, remodeling of membrane-bound systems, such as giant unilamellar vesicles, is finding applications beyond biological studies, to engineer cell-mimicking structures. Giant unilamellar vesicle fusion is rapidly becoming an essential experimental step as artificial cells gain prominence in synthetic biology. Several techniques have been developed to accomplish this step, with varying efficiency and selectivity. To date, characterization of vesicle fusion has relied on small samples of giant vesicles, examined either manually or by fluorometric assays on suspensions of small and large unilamellar vesicles. Automation of the detection and characterization of fusion products is now necessary for the screening and optimization of these fusion protocols. To this end, we implemented a fusion assay based on fluorophore colocalization on the membranes and in the lumen of vesicles. Fluorescence colocalization was evaluated within single compartments by image segmentation with minimal user input, allowing the application of the technique to high-throughput screenings. After detection, statistical information on vesicle fluorescence and morphological properties can be summarized and visualized, assessing lipid and content transfer for each object by the correlation coefficient of different fluorescence channels. Using this tool, we report and characterize the unexpected fusogenic activity of sodium chloride on phosphatidylcholine giant vesicles. Lipid transfer in most of the vesicles could be detected after 20 h of incubation, while content exchange only occurred with additional stimuli in around 8% of vesicles.

## 1. Introduction

The development of artificial cells based on phospholipid giant unilamellar vesicles (GUVs) has widened the application of these compartments beyond their role as membrane models [1]. GUV-based artificial cells must have access to a wide array of dynamic behaviours, such as the ability to host an internal metabolism [2], divide [3,4,5], aggregate in designed ways [6], and fuse [7,8,9,10,11,12]. Many implementations of these individual behaviors have been demonstrated, with the aim of integrating them to approximate the complexity of living systems. Among these, fusion procedures have drawn attention since they allow the transfer of water-soluble molecules to GUVs after their formation. Hemifusion with consequent lipid transfer has also garnered interest in the engineering of asymmetric membranes [13]. Phospholipid GUVs, once formed, are stable for weeks [14], making fusion a challenging task to perform without perturbation. The main force to overcome is the repulsion between the hydration layers that bind the phospholipid headgroups of neighbouring compartments, and the suppression of membrane undulations in the contact area [15,16]. In vivo, this is accomplished by proteins that mechanically restructure the membrane to facilitate the formation of the initial point of contact (fusion stalk), followed by hemifusion, and pore formation in the hemifusion diaphragm [17]. Techniques to induce fusion in artificial cells rely on vesicles formed by lipids of opposite charge [2,10], reconstituted fusogenic proteins [18,19,20], divalent cations [12,21], or lipidated complementary DNA strands [5,7,9,22,23,24].

As artificial cells gain prominence, rigorous quantification and evaluation of experimental reproducibility is becoming a pressing issue. Previous quantification approaches of vesicle fusion products relied on the manual inspection of few events, such as by observation of fluorophore exchanges between compartments in microscopy, coupled with fluorometric techniques to measure the average properties of large and small vesicles [5,9,25]. This is incompatible with both in-depth optimization procedures via high-throughput screening, and the evaluation of variability within and among experiments. Careful evaluation of variance not only impacts experimental reproducibility, but also relates to the ability of a system to respond to external stimuli [26] and may indicate critical features of an optimization strategy.

The rapid development of artificial cell research has prompted interest in the statistical analysis of GUV populations, with recent works tackling the problem from various angles. Flow cytometry was one of the first techniques applied to this end, but it is limited due to its lack of spatial resolution [27,28,29]. Imaging flow cytometry (IFC) provides significant improvements through the collection of widefield fluorescence imaging data for thousands of objects in a flow chamber [14,30,31]. Although valuable, IFC is not yet widely available, nor does it allow data acquisition for GUV in their native state and under controlled conditions. Microscopy is usually paired to flow cytometry to provide this complementary information, but without specialized methodologies, it can only be used to obtain datasets for tens to a few hundred vesicles. Automatic vesicle detection by image processing is challenging to implement accurately, since GUV populations have inherent heterogeneities influenced by the vesicle formation methodology. GUVs formed using gentle rehydration are mixed with multilamellar vesicles, lipid aggregates, and multivesicular compartments [32]. This is also true for gel-assisted rehydration with possible hydrogel inclusions [33]. Additionally, oil inclusions and contamination are likely to appear in vesicles formed by emulsion-based techniques [34]. These different species will appear as objects with different contrasts and fluorescence intensities if fluorescent lipid probes are included. This in turn complicates image analysis, since each field imaged may have different pixel threshold values, which may require user input for optimal detection. Moreover, several of the dynamic processes displayed by functional GUVs make the analysis more challenging. Division and metabolic activity induce morphological changes that deviate from the resting spherical state, while fusion and hemifusion occurs in aggregated vesicles. Due to these complications, flexible analysis pipelines are strongly desirable. A popular approach for the automatic segmentation of GUVs is the application of a circular Hough transform, which biases the detected result towards objects with a circular profile [35]. The recent work by van Buren et al. [36], represents an example of flexible GUV detection, where several detection strategies are implemented to ultimately allow the user to select the best one based on their specific sample and imaging strategy. Another approach to overcome the high variability in vesicle suspensions is pairing conventional image analysis with machine learning approaches. Lee and co-workers used the circular Hough transform to select vesicles suitable for classification with convolutional neural networks to thereby identify the membranes that presented phase separation [37].

In line with these advances, tools to statistically study GUV-GUV fusion are needed. Previous techniques approached the problem using fluorogenic reactions or protein expression, which are triggered following content mixing. Exchange of lipidic components has been monitored by changes in fluorescent lipid emission due to Förster resonance energy transfer [10,31]. Taking advantage of the size of GUV, microscopy, or IFC to confirm fusion, provides a convenient alternative assay based on the colocalization of two fluorophores, either encapsulated in the lumen (content markers), or localized on the membrane (lipid markers) of separate populations of GUV.

We present a flexible analytical pipeline for content and lipid exchange quantification in GUV populations based on high-throughput imaging. Sedimented vesicles in a sealed chamber are imaged with a motorized stage to record a large sample surface, containing thousands of GUVs. GUV images are elaborated with ImageJ to identify and label the compartments based on their membrane fluorescence. The only assumption made in this step is the presence of at least one channel related to the fluorescence of a membrane probe. Adaptation of GUV detection to brightfield or phase contrast images is straightforward, but we focused on fluorescence, since this can resolve hemifused and aggregated GUVs. Information on fluorescence channels for lipid or content markers, together with morphological parameters for single GUVs, are saved for statistical analysis. This is performed by scripts developed in the R programming language for statistical computing. Raw images were used by an R script to compute the correlation coefficient between pairs of channels as measurements of the colocalization of various probes. This method was then compared with IFC to validate the quantitative reliability of this approach in identifying content and lipid exchange frequencies, vesicle concentration estimates, and size distributions, highlighting not only the advantages, but also the possible blind spots and pitfalls of the method. Finally, this new approach was applied to the analysis of aggregated GUVs, reporting the fusogenicity of sodium chloride, which was previously thought to be non-fusogenic.

## 2. Results

### 2.1. Analysis Workflow

**Figure 1:**
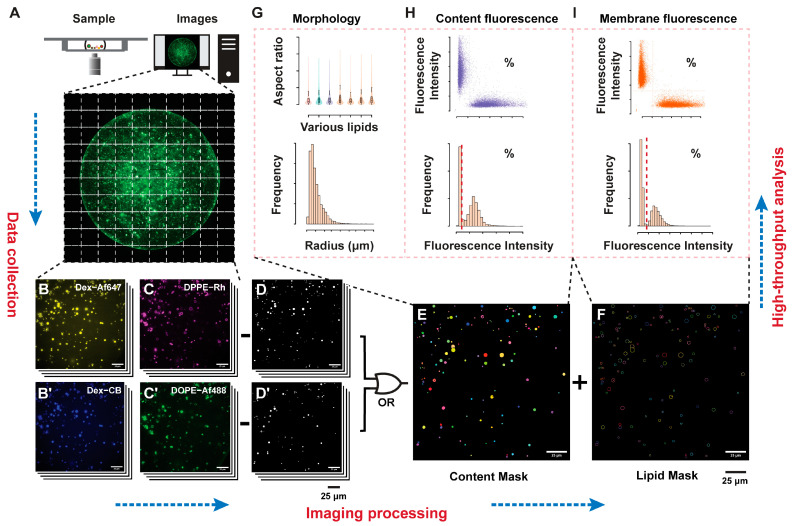
Schematic representation of the analysis workflow. Large image acquisition of the GUV samples in an imaging chamber (**A**) composed of multiple fluorescence channels related to content markers (**B**,**B’**), or lipid markers (**C**,**C’**), are acquired. Filtering (**D**,**D’**) and thresholding generates labelled content (**E**) and lipid (**F**) images, used to extract the morphological (**G**), content fluorescence (**H**), and membrane fluorescence (**I**) distributions that can be thresholded (dashed lines) to define the positive and negative GUV populations for each parameter.

GUV imaging data was acquired as multi-channel multi-point .nd2 files. For each image, labelled masks of content markers and lipid markers were produced by the ImageJ macro by sequentially filtering, thresholding, and combining the image channels (as schematically depicted in Figure 1). These masks were used to save fluorescence and morphology data for each GUV, and then were subsequently analysed in R. Details on filtering sequence and parameters are provided in sections A.1. Image processing details, and A.2. statistical analysis by R in the Supplementary Information SI. Object-wise fluorophore colocalization was evaluated in R by computing the correlation coefficient between two numeric vectors containing the pixel values of the GUV image in different fluorescence channels. The image was obtained by cropping a square window around the GUV of interest, with the option of selecting regions of interest within the window (lipid signal, content signal, or both). Colocalization in microscopy is commonly evaluated by the Pearson Correlation Coefficient (PCC) or the Manders Colocalization Coefficient (MCC) [38,39]. To optimize the accuracy of content and lipid exchange quantification, various correlation metrics were evaluated. The R correlation test routine implements correlation evaluation by PCC, Spearman’s coefficient, and Kendall’s coefficient. The latter are non-parametric correlation metrics not commonly used in colocalization studies but were tested nonetheless given the ease of implementation. Additionally, two thresholding methods for MCC were compared. The first considered the median signal in each channel as the threshold, and the latter the minimum fluorescence intensity detected in the GUV. Since the two markers being analyzed in these routines stain equivalent structures, only one of the two possible values of MCC for each pair of channels was considered. Populations of GUV positive for each fluorescent marker and for content/lipid exchange were defined by automatic thresholding. The R package multimode was used to find the antimode of bimodal distributions of fluorescence and correlation. These can be set on bimodal distributions obtained by acquiring the datasets of mixed GUVs, or by constructing synthetic datasets by random sampling of a negative and positive dataset. For correlation thresholding, random sampling was found to be more reliable, and was optimized by evaluating the accuracy and threshold values at different ratios of positive to negative vesicles. After defining the GUV populations, the scripts output the concentration estimates and relative percentages of GUVs in each population. Positive objects were visualized by producing montages of single GUV paired with single channels images. The portion of positive GUVs with the lowest correlation score (based on the positive percentage in negative samples) was considered false positive and visualized in the same way to assess the analysis performance. The workflow of the analysis and the possible outputs are depicted in Figure 1, Supplementary Figures S1 and S2.

**Figure 2:**
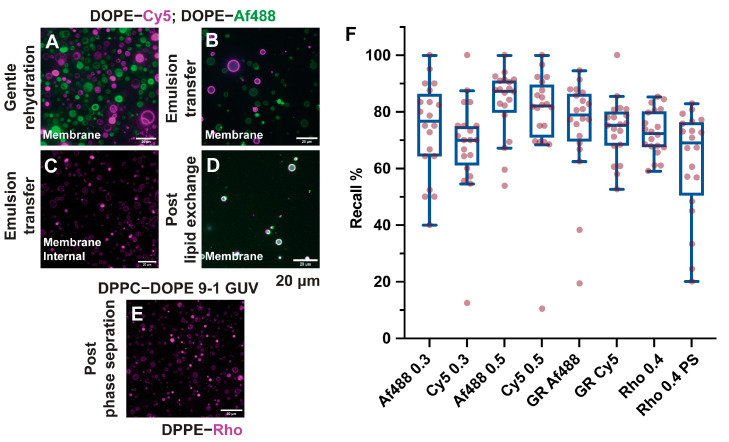
Recall analysis for heterogeneous GUVs. Object recall for a variety of GUVs preparations was evaluated. Samples prepared by gentle rehydration, containing two different fluorophores and multilamellar or multivesicular vesicles, (**A**) showed highly variable recall scores (boxplot F, label GR Af488, and GR Cy5). GUVs prepared by emulsion transfer with DOPE–Cy5 and DOPE-Af488 ((**B**) on separate populations at 0.6%, (**D**) on the same vesicles but at 0.3% concentration) and DPPE–Rh ((**C**) at 0.4%) can contain oil inclusion, and show a more consistent recall score, with values proportional to the amount of fluorescent lipids used ((**F**) boxplots labelled as Af4880.3, Cy5 0.3, Af488 0.6, Cy5 0.6, and Rho 0.4). Phase segregation in bilayers can lead to uneven fluorescence distributions in the membranes, as depicted in (**E**) for the DPPC–DOPE 9-1 GUV stained with 0.4% DPPE-Rh. This affects recall, making it more variable on an image-to-image basis ((**F**) boxplot label Rho 0.4 PS).

The ImageJ filtering sequence was chosen and optimized to be robust to the fluorescence variability in the samples. GUV are heterogeneous, and this heterogeneity strongly depends on the method used to produce the compartments. Rehydration methods yield multilamellar or multivesicular compartments, and these membranes can present oil inclusions and solvent contaminations when produced by emulsion transfer or microfluidic methods. When lipid markers are included, all these contaminations change the fluorescence profile of the GUV membranes, making GUV detection sample-based, and highly variable between images. Additionally, lipid phase separation can lead to an uneven distribution of fluorophore-tagged lipids. The recall (as defined in [36]) of GUV detection prepared by gentle rehydration and emulsion transfer with different fluorophores, and fluorophore concentrations is presented in Figure 2. The F1 score (defined as the harmonic mean of precision and recall, commonly used to quantify object detection performance) for the same experiments is also presented in Supplementary Figure S3. Since no false positive detections were found, the recall analysis is more relevant than the F1 score to quantify object detection reliability. All conditions tested yielded an average recall score of between 0.8 and 0.75, which is on par with other automated GUV detection methods [36]. A larger field-to-field recall variability was noticeable in samples prepared by gentle rehydration and samples with phase-separated GUV.

### 2.2. Imaging Setup Optimization

**Figure 3:**
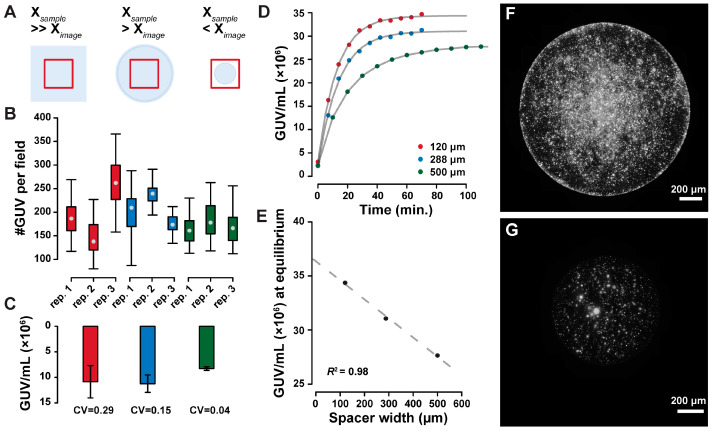
Imaging setup optimization. By varying the ratio of imaged area to sample area (**A**), the field-to-field variability of detected GUVs can be minimized (**B**), leading to more accurate GUV concentration estimates (by the coefficient of variation CV in (**C**)). Imaging of GUVs during their sedimentation shows faster equilibration in thinner imaging chambers ((**D**), where the dots represent the measurements and the lines represent asymptotic exponential fits), with linear dependency of the equilibrium concentration on the spacer width ((**E**), dots fitted values for growth limit in (**D**), dashed line linear fit with *R*^2^ = 0.98). Sample evaporation affects the distribution of GUVs over the imaged surface, as shown in the imaging chamber filled with air (**F**), or a fluorinated oil (**G**).

Imaging of free-standing GUVs can be challenging, motivating the use of various immobilization techniques [40,41]. To image the GUVs in their native state, we did not immobilize the compartments in the imaging chamber. GUVs spontaneously collected at the bottom of the imaging chamber by sedimentation due to the different densities of encapsulated droplet solution (DS), and external hosting solution (HS). This technique is commonplace but, to our knowledge, has not been used to quantify GUVs in a suspension. Different imaging setups were characterized by evaluating the field-to-field variability and concentration estimate in triplicate acquisitions of the same sample when the imaged area was a small part, on the same scale, or a larger part, of the sample’s area. As summarized in Figure 3A–C, the sample-to-sample variability was minimal when the GUVs suspension was entirely included in the imaged area. This may be due to uneven sedimentation, leading to GUVs density variation across the area. Timelapses of GUVs sedimentation were used to evaluate the appropriate incubation time for complete equilibration. Spacers of varying thickness were used to highlight different sedimentation timescales depending on the sample volume. The sedimentation time depends on sample thickness, as depicted in Figure 3D. The concentration estimate at equilibrium also depends on the sample thickness with a linear relationship (Figure 3E). GUVs can sediment unevenly over the coverslip either due to convection, or through osmotic imbalances due to sample evaporation [6]. This uneven GUVs distribution is depicted in Figure 3F and can be minimized by suppressing evaporation as depicted in Figure 3G, after filling the imaging chamber with fluorinated oil to isolate the samples.

Sample preparation was critical to ensure the reproducible evaluation of GUVs concentration. Nevertheless, the quantification of GUVs in a sealed imaging chamber with a defined thickness was deemed unreliable due to the incomplete sedimentation of the particles even under long timescales, when the vesicles on the bottom of the chamber reached a dynamic equilibrium with those that were out of focus.

### 2.3. Quantification of Vesicle Subpopulations

The accuracy of GUV classification in positive and negative populations for content/lipid exchange was evaluated as described in Section 4.4, to optimize the parameters involved in object-wise fluorophore colocalization measurements. Briefly, datasets for microscopy and IFC were constructed by recording triplicate measurements of the stained vesicles as described in Table 1. Content exchange experiments involved 1,2-dipalmitoyl-sn-glycero-3-phosphoethanolamine-Rhodamine (ammonium salt) (DPPE-Rh) as the lipid marker, Dextran-Alexa Fluor™ 647 (10,000 MW, anionic, fixable, Dex–Af647), or Dextran-Alexa Fluor™ 488 (3000 MW, anionic, Dex–Af488) as content markers. GUVs in lipid exchange experiments were stained using 1,2-dioleoyl-sn-glycero-3-phosphoethanolamine-N-(TopFluor^®^ AF488) (ammonium salt) (DOPE–Af488), or 1,2-dioleoyl-sn-glycero-3-phosphoethanolamine-N-(Cyanine 5) (DOPE–Cy5) as lipid markers, and Dextran–Cascade Blue™ (10,000 MW, anionic, lysine-fixable, Dex–CB) as content marker. The datasets contained samples of the isolated P1, P2, and PC populations, and mixed samples containing various ratios of all three populations. Specifically, NC contained 1:1 P1 and P2, while M1 to M4 contained P1:P2:PC at 1:1:1, 1:1:0.5, 1:1:0.1, and 1:1:0.05, respectively. Analysis parameters for microscopy were optimized by screening a variety of values. For each one, false-positives were evaluated by counting negative GUVs in a sample of 121 positive vesicles. Similarly, false negatives were counted in a sample of 121 positive GUVs. Window width, use of masks, correlation coefficient, and thresholding parameters were sequentially optimized by choosing the parameter that yielded the lowest false-negatives and false-positives. The data shown for MCC refers to the correlation of the Dex–Af647 signal relative to the Dex–Af488 signal. The results of each optimization are shown in Supplementary Figure S4 (lipid exchange), and Figure S5 (content exchange). The correlation score between the fluorescence channels was influenced by the GUVs neighbourhood, as depicted in Supplementary Figure S6. For this reason, the GUVs density should be controlled with care during sample preparation, by appropriately diluting the GUVs suspensions prior to visualization. This dependency can be abolished by using masks when evaluating correlation (Figure S6B,D), but this makes the negative and positive control populations less separated, leading to higher rates of classification errors (Figure S6G–I). Samples with excessive GUVs density may still be analyzed by this method, albeit with trade-offs in accuracy due to mask use, as reported in Supplementary Figures S4 and S5.

To validate the quantification of GUVs content and lipid exchange by the analysis method developed here, dilutions of positive control (PC) GUVs mixed in various ratios with P1 and P2 GUVs were acquired on the same day by microscopy and IFC.

**Figure 4:**
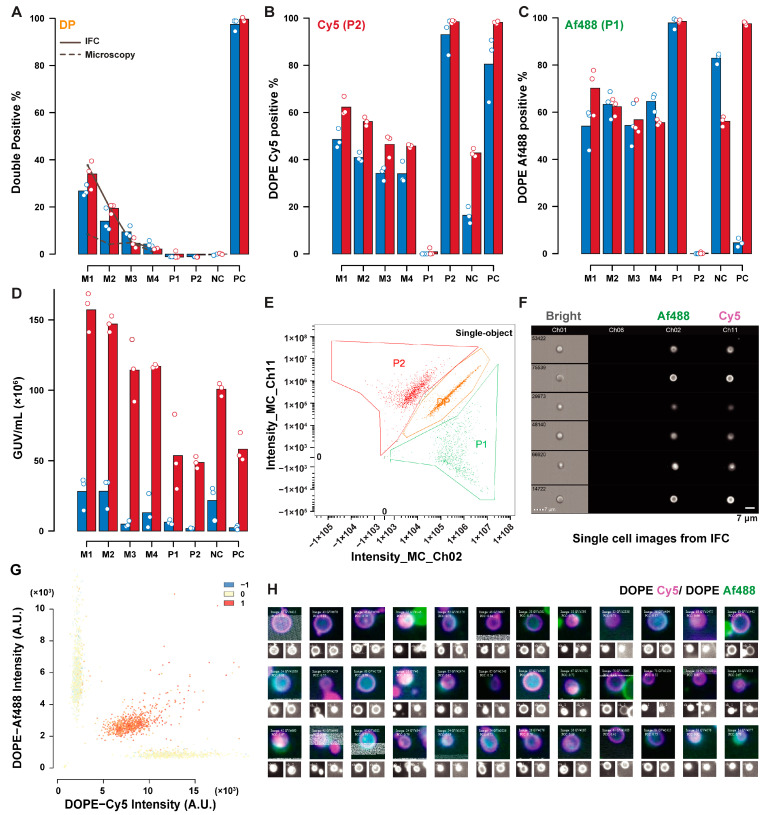
Comparison of IFC and microscopy analysis of the lipid exchange controls. Comparative results for percent estimates of DP GUVs (**A**), DOPE–Cy5 positive (**B**), DOPE–Af488 positive (**C**), and concentration estimate (**D**) obtained by microscopy (red), and IFC (blue). Datasets contained triplicate readings of P1 (DOPE–Af488 positive); P2 (DOPE-Cy5 positive); PC (containing both DOPE–Af488 and DOPE–Cy5); and NC (P1 and P2 1:1); and mixed samples of P1:P2:PC named M1 (1:1:1); M2 (1:1:0.5); M3 (1:1:0.1); and M4 (1:1:0.05). The solid line in (**A**) represents the expected double-positive percentage based on the GUVs concentration estimates from IFC, while the dashed line represents the same quantities using microscopy-based concentration estimates. An example of a fluorescence dot plot for a mixed sample is depicted in (**E**) (IFC, with an extracted sample of double-positive GUVs in (**F**)) and (**G**) (microscopy, with color representing the correlation score). Selected objects within a population can be displayed in panels composed of the merged image with its single channel representations below, as shown for DP GUVs in (**H**)).

Figure 4 summarizes the comparative results for lipid exchange measured by IFC (red), and microscopy (blue). Differences in the percentage of GUVs positive to DOPE–Cy5 (P2), and DOPE–Af488 (P1) are a result of the different definitions of the two populations in IFC and microscopy. In the former technique, P1 and P2 were defined as the sum of double positive (DP), and P1 or P2 polygonal gates respectively, as depicted in Figure 4E (with a sample of DP-gated GUV in Figure 4F). The definition for microscopy was instead based on the automatic thresholding of the fluorescence histogram in a negative control sample, with a 1:1 volumetric ratio of P1 and P2. This thresholding may be influenced by the relative abundance of the two populations, fluorescence influence of neighboring vesicles on each other, and the background from out-of-focus GUVs, acquired by pinhole crosstalk in spinning-disc confocal microscopy. For this reason, thresholding on a mixed sample was preferred to thresholding on a synthetic dataset, which may underestimate noise due to these contributions in the real samples.

Additionally, sample sizes may affect the accuracy of thresholding, especially in the dataset here, where the GUVs number in some controls tended to be lower (sample sizes for the various datasets are summarised in Table S1). The GUV concentration estimates show that microscopy underestimates the GUV concentration compared to IFC. The double-positive percentage (representing lipid exchange-positive GUVs) demonstrated a good agreement between the two techniques. The double-positive estimate based on the GUVs concentration and the volume ratio of PC GUVs added in each mixed sample is represented with the lines in the bar plot. IFC estimates (solid line) are consistent with the measurements, while the microscopy estimates (dashed line) suffer from the low accuracy of concentration estimates. Figure 4G shows the fluorescence dot plot with correlation score presented in colored dots from microscopy. GUVs can be selected based on their properties and can be shown in image panels composed of single GUVs images, as shown in Figure 4H for a sample of DP GUVs.

**Figure 5:**
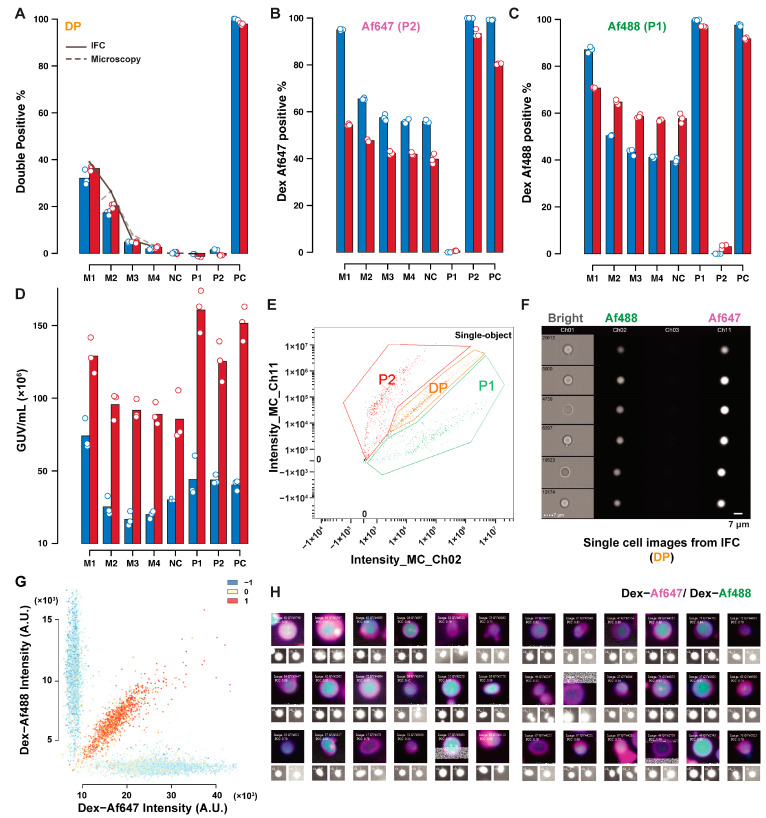
Comparison of IFC and microscopy analysis for content exchange. Results of IFC (red) and microscopy (blue) analysis of mixed GUVs samples. Percentages of double-positive GUV (**A**), Dex–Af647 positive (**B**), Dex–Af488 positive (**C**), and GUVs concentration estimates (**D**) are reported. The dashed line in (**A**) represents the expected percentage of double-positive GUVs based on the concentrations measured by IFC (solid line), and microscopy (dashed line). A fluorescence dot plot of a mixed sample is reported in ((**E**), IFC), and in ((**G**), microscopy), with samples of double-positive GUVs in ((**F**), IFC) and ((**H**), microscopy).

Figure 5 depicts the IFC-microscopy comparison for the content exchange mixed dataset. Thresholding of P1 and P2 yielded consistent trends in P1 and P2 identification between these two techniques (Figure 5B,C). An offset due to different classification criteria is, however, visible. The same tendency to underestimate the GUVs concentration by microscopy measurements was observed in Figure 5D, with an average ratio between microscopy and IFC estimates of 0.31. Double-positive GUVs percentages were consistent, both between techniques, and with expected values based on GUVs concentration, indicating that part of the inconsistencies reported in the lipid exchange dataset were due to the sample size, as Figure 5 represents much larger samples. The sample M1 is a notable outlier in this discussion, due to the excessive GUVs density in this sample (Supplementary Figure S7), leading to different background fluorescence levels. Despite this, the DP percentage is still consistent, as the correlation does not depend on fluorescence levels, but only on the covariance and variance of the two signals. The comparison between IFC and microscopy highlights the robust quantification of GUVs both in the content and lipid exchange controls, even when other estimates and quantifications (concentration, single positive GUVs) show less consistency, highlighting the robustness of the approach. The different results obtained for lipid exchange and content exchange indicate that sample sizes between 1000 and 15,000 objects were optimal for quantification with the area imaged in our setup, to avoid the amplification of stochastic fluctuations and overcrowded samples. A summary of fluorescence and size distributions obtained from the two techniques are available in Supplementary Figures S8 and S9 for lipid and content exchange datasets, respectively. A significant difference in the average GUVs radius between the two techniques was observed, with larger sizes reported by IFC. Masks for radius estimate in IFC were defined by content fluorescence and may include pixels outside of the GUVs (Supplementary Figure S10). On the other hand, microscopy measures size based on the lumen, resulting in lower estimates of the compartment size. Additionally, size estimate from microscopy involve compensation based on the axial offset used during acquisition (discussed in the Appendix A.1, and depicted in Supplementary Figure S11), and may lead to the generation of skewed results if this offset is not estimated correctly.

### 2.4. Quantification of Content Exchange and Lipid Exchange Efficiencies in Aggregated GUVs

Having assessed the performance of the analytical approach, we applied it to the characterization of lipid exchange and content exchange following GUVs aggregation through sodium chloride addition. GUVs are kept separated from one another by their zeta (ζ) potentials, which are present even when composed of zwitterionic lipids due to the specific orientation of the headgroups dipoles [42]. Electrolytes such as sodium chloride screen this potential, leading to GUVs aggregation. This aggregation has been used to control GUVs-GUVs association architectures in the past [43], showing no fusogenic activity of monovalent cations. Salt gradients were also shown to have a deep impact on membrane properties by associating with phospholipid headgroups, and changing their molecular area, leading to increased membrane rigidity, tension, and negative spontaneous curvature [44,45]. We found that after long-term incubation (20 h), frequent lipid exchange events could be observed among the GUVs with a variety of lipid compositions (Supplementary Figure S12). Fluorophore distributions characteristic of hemifusion [46] were observed, highlighting differences between adhering and hemifused GUVs depicted in Supplementary Figure S13. After aggregation and hemifusion, GUVs can be separated by washing the sedimented vesicles with HS, making visualization and quantification possible.

**Figure 6:**
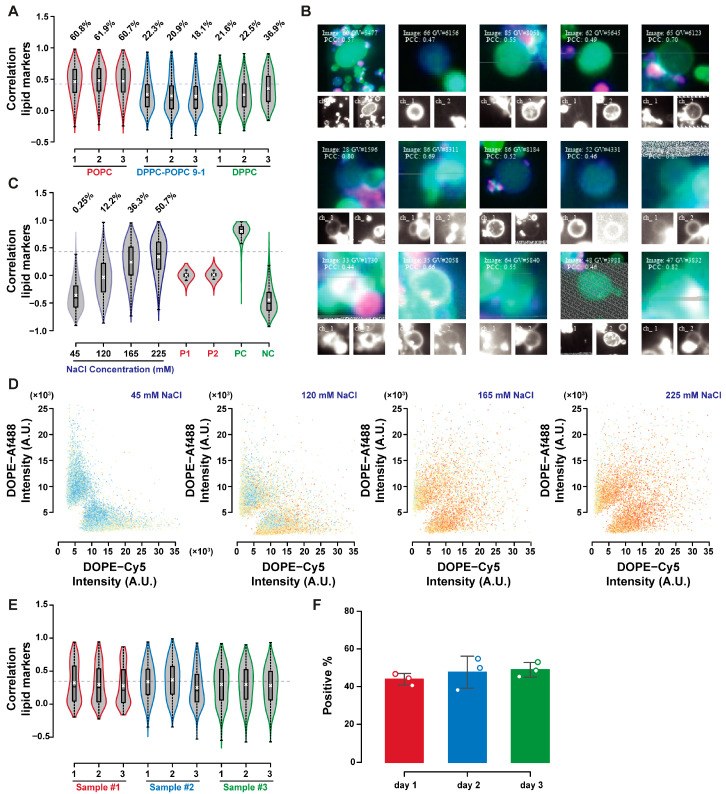
Effect of sodium chloride incubation on lipid exchange. Lipid exchange is influenced by lipid mobility, as depicted by correlation distribution shifts in POPC, DPPC–POPC 9-1, and DPPC GUVs after incubation in NaCl 225 mM. (**A**) The positive events isolated from the POPC sample not only display lipid transfer, but also aggregation; PC and NC refer to the positive and negative controls as defined in the previous sections. (**B**) The correlation shift is proportional with the sodium chloride concentration, (**C**) with mixing of the lipid markers signals and correlation increasing with NaCl concentration (**D**). Lipid exchange by overnight aggregation in sodium chloride solutions was reproducible, as depicted by the correlation distributions for experiments carried out on three different occasions, (**E**) and the average percentage of GUVs above the correlation threshold (**F**).

Characterization of lipid exchange in phosphatidylcholine GUVs is shown in Figure 6. Lipid transfer was more frequent in POPC GUVs compared to DPPC or DPPC–POPC 9-1 GUVs when incubated at room temperature overnight (after mixing at 55 °C and then cooling to 25 °C at 0.5 °C/min, in 225 mM sodium chloride, Figure 6A). GUVs above the correlation threshold are shown in Figure 6B, highlighting the impact of GUVs aggregation in lipid exchange detection. GUVs may be considered positive if the isolated objects can present both lipid markers, or if the neighbouring positive GUVs can push the score of the GUVs of interest above the threshold despite it being negative itself. This potential double counting does not prevent reliable quantification in the control experiments, as discussed in the previous section. Part of the signal detected as lipid exchange may still be due to aggregation, so its quantification requires better GUVs-GUVs separation, or better optical sectioning. Figure 6C shows the shift in correlation distribution with increasing sodium chloride concentration in POPC GUVs, paired with the change in fluorescence distribution with the mixing of the two fluorophores signals in Figure 6D. The reproducibility of lipid exchange by these means was evaluated by repeating the experiment in triplicate on three different days. The emulsion transfer method can have large batch-to-batch variabilities, making reproducibility assessments even more critical in GUVs studies. Figure 6E allows the comparison of correlation distributions over the triplicate experiments performed over three days, highlighting their similarity. The percentage of GUVs above the correlation threshold did not vary in the three replicates, as shown in Figure 6F (with no statistically significant differences having been detected by the analysis of variance).

**Figure 7:**
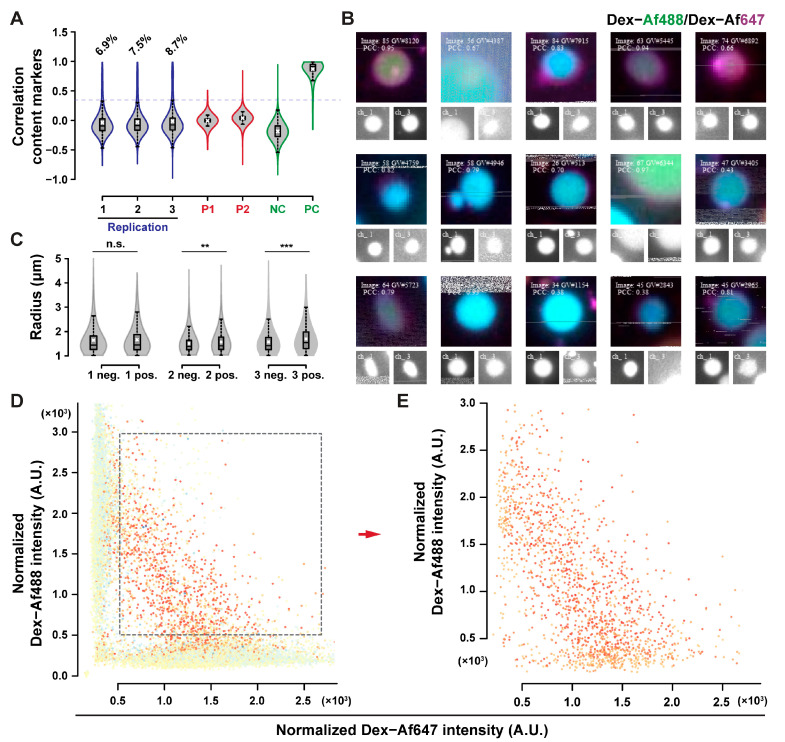
Content exchange upon sodium chloride incubation. (**A**) Content exchange between MPPC–DOPE 9-1 GUVs as indicated by shift in correlation; PC and NC refer to the positive and negative controls as described previously. Percentages of objects above the correlation threshold are reported as 6.9%, 7.5%, and 8.7%, respectively. (**B**) Extracted images of positive GUVs. The radius of the positive GUVs was slightly larger than the rest of the population in two of the three samples assessed (**C**). Positive GUVs had negatively correlated fluorescence signals, (**D**,**E**) indicating dilution of fluorescent probes upon exchange.

No content exchange could be detected from the qualitative observation of POPC GUVs micrographs. MPPC-DOPE 9-1 was used to assess content exchange since less aggregates persisted after HS washing with this composition. Additionally, the transition of MPPC to the liquid-ordered state, and de-mixing of DOPE can provide membrane tension, which is known to facilitate content exchange in hemifused systems [47,48,49,50]. MPPC-DOPE 9-1 GUVs were incubated in 225 mM sodium chloride at 37 °C, above the main transition temperature of MPPC (with transition in mixed systems at lower temperatures, evaluated by the ζ potential shift [42], as depicted in Figure S14) for 20 h, and then cooled at room temperature. Results for the content exchange experiment are presented in Figure 7. Around 8% of the GUVs were above the correlation threshold (Figure 7A), with a good accuracy in positive GUVs classification (Figure 7B). Positive GUVs exhibited a significantly larger than average radius in two of the three replicates (evaluated by the one-tailed Wilcoxon test), although the difference is on the scale of 0.1 µm, which is smaller than the expected difference for the size of GUVs after complete fusion. The significance of the test is most likely due to the large sample size involved, rather than due to an actual size difference [51]. GUVs with both fluorophores had a specific fluorescence distribution, as indicated in the fluorescence dot plots (Figure 7D,E). The negative correlation between the two fluorescence intensities after content exchange may be explained by a simple model of random content transfer between hemifused GUV (see the Appendix A.4. Simulated Datasets section in the Appendix A for further details).

Overall, GUVs-GUVs fusion conditions lead to the production of heterogeneous samples via aggregation, hemifusion, mixing, and dilution of various probes. The optimal strategy identified in the previous sections was applied to these highly heterogeneous conditions, highlighting the differences between the control samples used previously, and a real-case scenario. Nevertheless, quantification of these fusion products was shown to be reliable both in terms of lipid and content exchange, with some caveats concerning the sensitivity of lipid exchange detection to aggregation.

## 3. Discussion

We present a novel strategy to quantify lipid and content exchange in GUVs using high-throughput image analysis. Large sample sizes and robust statistical analysis permitted the application of optimization strategies to GUVs experiments. Analysis of GUVs usually involves a few handpicked vesicles from the same population to extrapolate results, yielding large uncertainties, and leaving doubts on their reproducibility. With the method we developed here, the correlation between the fluorescent signals in the membrane and the lumen of GUVs provided a robust criterion for the detection of fusion products. We applied colocalization analysis to measure content exchange and lipid exchange in GUVs fusion experiments, first by validating the method in control experiments using IFC, and then by measuring lipid and content exchange in aggregated GUVs. By analysing large populations of GUVs, it was possible to optimize the imaging conditions for quantification, and to characterize GUVs sedimentation in a sealed imaging chamber. After optimization of the analysis parameters, our microscopy-based approach yielded results that were in quantitative agreement with IFC, which is the current state-of-the-art for the in-depth characterization of fluorescence in large populations of GUVs. GUVs concentration estimates were consistently lower in microscopy. Loss in cases of recall lower than one, out of focus GUVs, and vesicles excluded from analysis due to them lying on image boundaries, all contributed to this discrepancy. Better characterization of each contribution may lead to a compensation factor between the two techniques, thereby accounting for these losses, and yielding accurate GUVs concentration estimates. The approach described here was designed to work under a variety of conditions and with samples of heterogeneous membranes, by applying local contrast enhancement and thresholding. We demonstrate that GUVs detection was robust with respect to the fluorophore type and concentration, and to the method used to make GUVs. No GUVs shape assumption was made, aside from a convexity threshold, which was set to exclude noise generated from empty images. If no empty images are included in the acquisition, this shape filtering step can be removed as well, leading to unbiased detection of GUVs with a variety of shapes. The analysis of multi-channel images of GUVs was designed to be flexible, with the ability to analyze any number of fluorescent channels. This can be applied to multiple content or lipid marker transfer events, or to design a joint lipid-content exchange assay by carefully selecting fluorescent probes. With the appropriate controls, this method could quantify GUVs fusion, hemifusion, leakage, and lysis in one experiment. Establishing relationships between multiple fluorescence signals and their colocalization may also be of use in studies of GUVs hosting complex reactions or expressing multiple gene products, thereby providing valuable tools to characterize individual GUVs as reaction vessels.

Lipid and content exchange between the fusing GUVs were quantified for a variety of lipid systems upon sodium chloride-induced aggregation. No fusogenic activity of monovalent cations on phospholipid GUVs has been reported thus far, probably due to the long timescales involved in the process. Compared to other fusogens, such as calcium ions, the activity of sodium chloride was weak. Long incubation times and large concentration gradients were required to observe appreciable effects. In non-fusogenic compositions, such as pure POPC, only lipid exchange was observed, while content exchange required other inputs such as temperature–sensitive lipid compositions. With the tools presented here, hemifusion and fusion of GUVs using other alkali halides, lipid compositions, and osmotically induced membrane tension can be easily screened and utilized to optimize a protocol for efficient GUVs-GUVs content exchange and lipid exchange. We found indications that content exchange upon the incubation of MPPC–DOPE 9-1 GUVs with sodium chloride was due to the partial exchange in the hemifusion intermediate, rather than full fusion. The lack of full fusion may not be an issue in artificial cell applications, as it can be used to deliver contents between compartments while also maintaining size homeostasis without the need for division. Lipid and content transfer have been used, up to now, as a proxy for vesicle fusion, but the results presented here indicate that transfer of these materials may not be univocally linked to the full fusion of GUVs. Further characterization of GUVs-GUVs interactions is required to describe these fusion events.

Whether or not morphological data based on a single GUVs image can be used to extract meaningful information is still an open question. We performed size compensation on GUVs radii to account for the fact that the compartments were not imaged on their equatorial plane (Supplementary Figure S11) but did not carry out further morphological characterization. GUVs deformation induced by osmotic deflation can be used to change their shape and evaluate whether the data generated by our methodology can be used to perform quantification of phenomena relevant to GUVs division in addition to GUVs fusion. Nevertheless, the advancements we present are required steps that have now been taken towards the characterization of GUVs fusion-division cycles for the development of dynamic and self-sustaining artificial cells.

## 4. Materials and Methods

1-palmitoyl-2-oleoyl-glycero-3-phosphocholine (POPC), 1-myristoyl-2-palmitoyl-glycero-3-phosphocholine (MPPC), 1,2-dipalmitoyl-sn-glycero-3-phosphocholine (DPPC), 1,2-dioleoyl-sn-glycero-3-phosphoethanolamine (DOPE), 1,2-ditridecanoyl-sn-glycero-3-phosphocholine (13PC), and 1,2-diphytanoyl-sn-glycero-3-phosphocholine (DPhPC) were purchased from Avanti Polar Lipids (Birmingham, AL, USA), either in powder or chloroform solution. They were dissolved in chloroform (Sigma, St. Louis, MO, USA) and used without further purification. Membranes were fluorescently marked with the fluorescent-tagged lipids 1,2-dioleoyl-sn-glycero-3-phosphoethanolamine-N-(TopFluor^®^ AF488) (ammonium salt) (DOPE–Af488), 1,2-dipalmitoyl-sn-glycero-3-phosphoethanolamine-Rhodamine (ammonium salt) (DPPE-Rh), and 1,2-dioleoyl-sn-glycero-3-phosphoethanolamine-N-(Cyanine 5) (DOPE–Cy5), which were all purchased from Avanti Polar Lipids (USA) as well (except for DPPE-Rh, which was acquired from Thermo Fisher, Waltham, MA, USA). All water-based solutions were prepared with MilliQ water purified with a purelab flex elga system. Glucose, sucrose, sodium hydroxide, hydrochloric acid, and sodium chloride were purchased from Sigma. N-(2-Hydroxy ethyl)-Piperazine ethane Sulfonic acid (HEPES), Dextran–Cascade Blue™ (10,000 MW, anionic, lysine fixable, Dex–CB), Dextran-Alexa Fluor™ 647 (10,000 MW, anionic, fixable, Dex–Af647), and Dextran-Alexa Fluor™ 488 (3000 MW, anionic, Dex–Af488) were all obtained from Thermo Fisher. Novec 7500 engineered fluid used in sample preparation was bought from 3M. Mineral Oil (light, BioReagent, Sigma M5904) and squalene (TCI) were used as lipid carriers for the formation of the GUVs. PlusOne Repel Silane ES (Cytiva) was used following the manufacturer’s instructions to treat the microscopy slides, making them hydrophobic.

### 4.1. Vesicle Preparation

GUVs were prepared by the emulsion transfer method (ET) and gentle rehydration (GR) to compare the performance of vesicle segmentation under various conditions. The emulsion transfer method was modified from our previous work [14]. A chloroform stock solution of lipids was dried in a glass vial under nitrogen flow and for 40 min in a vacuum chamber. The dry film was solubilized in light mineral oil (POPC GUVs), or squalene (all other compositions) by heating at 80 °C for 10–20 min at a final concentration of 0.6 mM. A total of 200 µL of the lipid carrying oil were layered on 200 µL of a glucose solution (glucose 500 mM, HEPES pH 7.2 50 mM; HS), and incubated until the interface between the two phases flattened. Following this, 200 µL of lipid carrying oil were emulsified with 10 µL of droplet solution (sucrose 500 mM, HEPES pH 7.2 50 mM; DS), poured in the tube containing oil and HS, and centrifuged at 500× *g* for 10 minutes. GUVs were completely pelleted by centrifuging at 5000× *g* for 3 min, following which the pellet was washed once by resuspension in 200 µL of HS in a new tube and centrifuged at 5000× *g* for three minutes. Based on the experiment carried out, fluorescent DS were prepared by adding Dex–Af488 (at a final concentration of 40 µM); Dex–Af647 (at a final concentration of 10 µM); and Dex–CB (at a 40 µM of final concentration). All experiments were conducted with POPC GUVs containing a small fraction of fluorescent lipid: 0.4% mol/mol DPPE–Rh for content exchange experiments, and 0.6% mol/mol of DOPE–Af488 minor DOPE–Cy5 for lipid exchange experiments. To test the segmentation performance with phase-separated membranes, GUVs were prepared with a mix of 90% DPPC and 10% DOPE. The protocol was modified to work above the liquid-ordered to liquid-disordered transition temperature of DPPC by carrying out the incubations at 55 °C and performing all centrifugation steps at 40 °C with a force of 16,100× *g* for 1 min. Vesicles were cooled down to room temperature at a rate of 0.5 °C per minute. The same protocol was adopted to prepare MPPC-DOPE 9-1 GUV for the content exchange experiments. Gentle rehydration was performed following [34] by drying a 4 mM lipid in chloroform stock solution on a roughened Teflon support at 60 °C for 2 h. The film was placed in a glass vial and pre-hydrated at 60 °C by placing the vial in a container partially filled with MilliQ water for 4 h. Vesicles were allowed to form over night at room temperature by adding 5 mL of swelling solution (DS) and washed in HS to an appropriate final concentration.

### 4.2. Microscopy

Vesicle samples were imaged by placing a small volume of suspension on a glass slide (treated with Repel Silane) with an adhesive imaging spacer (500 µm thick Press-to-Seal™ Silicone Isolator, Grace BioLabs; 288 µm thick FrameSeal in situ PCR spacers 65 µL, BioRad (Hercules, CA, USA); 120 µm thick SecureSeal imaging spacers, Grace BioLabs). The imaging chamber was sealed with a silanized 1.5H coverslip (22 * 22 mm Marienfield), and vesicles were allowed to sediment by inverting the chamber and incubating for at least an hour unless otherwise specified. To limit evaporation and ensure the homogeneous coverage of the slide’s surface, the chamber was filled with fluorinated oil Novec 7500, ensuring no air was trapped with the samples. Imaging was carried out with a Nikon Ti2 microscope equipped with a CREST Optics X-Light V2 Spinning Disk module. Samples were imaged with a Plan Apochromatic 60 × Oil (NA 1.4), and the images were recorded by an Andor iXon Ultra 888 EMCCD camera. Monochromatic light was provided by a Lumencore SpectraX light engine equipped with several LEDs paired with dichroic beam splitters. Emission from various fluorophores was collected through bandpass filters paired with the appropriate excitation light (configuration available in the Supplementary Table S2). Acquisition routines were set up using Nikon AR Elements software. Each sample was imaged in a 10 by 10 square composite using the microscope’s perfect focus system (PFS) to keep the imaging plane at a fixed distance from the coverslip (2 µm). The output was set as a multipoint .nd2 file, containing images indexed by snake-by-rows ordering. No overlap between neighbouring frames was acquired, although the ImageJ macro allows cropping by a set overlap fraction if needed.

### 4.3. Image Analysis

A complete description of the scripts used in this work is available in the Appendix A.1. (Image processing details) and Appendix A.2. (Statistical analysis by R). After GUVs segmentation, the ImageJ macro saves contrast-adjusted jpeg images for the quick visualization of label maps, merged, and single channel images. In a second folder, csv files for morphology and fluorescence analysis were saved together with the raw images in tiff format for every channel and mask and used by the R script to compute correlation scores. GUVs data are imported in R, and background subtraction is performed in every fluorescence channel (whether content or lipid related) by subtracting the average minimum pixel fluorescence for each channel different from the one being elaborated. Correlation was evaluated among pairs of related channels on every GUVs by importing its images in tiff format, and then cropping them in a square window centered on the GUVs centroid. The pixel values of the tiff images were then converted into numeric vectors, and a correlation test was conducted to compute a correlation score. Different modalities for the correlation test can be selected among the default ones provided in the R environment. The Manders colocalization coefficient was computed in a separate function, according to [38]. The threshold values for the pixel intensities were set by taking the median pixel value in the square window, or by setting it to the minimum pixel intensity within the GUVs. After colocalization evaluation, thresholding of the fluorescence and correlation values was performed to define the populations positive to each content or lipid marker, and to exchanges in fluorophores. This was done automatically by finding the antimode of bimodal distributions of fluorescence or correlation using the multimode R package [52]. This can be applied to distributions that were obtained from mixed samples, or by constructing mixed datasets through the random sampling of existing ones. Finally, a series of data visualization steps were performed to output histograms of fluorescence intensity, aspect ratio, radius and correlation, fluorescence dot plots, and violin plots for each sample. To further visualize colocalization in relation to GUVs fluorescence, dot plots with color-coded points based on correlation value were also saved. Panels of GUVs images positive for lipid or content exchange were produced by random sampling of the various datasets. False-positive panels for each sample were also produced by sampling a number of GUVs that were proportional to the percentage of positive events in negative control samples. These were then subtracted from the final content or lipid exchange percentage.

### 4.4. Evaluation of Analysis Performance

Recall was evaluated by selecting 20 random images from the 100 images of each tested condition. The number of distinct visible compartments that did not intersect the image border was counted manually in each one, and the ratio between the GUVs detected by the ImageJ macro and by manual detection was the recall score for that image. To optimize the computation of correlation scores, window width, mask use, type of score, and threshold setting were all evaluated. A dataset with triplicate readings of four mixed samples with GUVs in various ratios was constructed (P1-P2-PC M1: 1-1-1; M2: 1-1-0.5; M3: 1-1-0.1; and M4: 1-1-0.05 volumetric ratio). For each of the 12 datasets, a panel of 121 GUVs was randomly sampled from the positive and negative portions of the dataset. The GUVs that did not exhibit fluorescence in both channels in the positive sample were considered false-positives, while GUVs fluorescence observed in both channels but drawn from the negative population were considered as false-negatives. The parameters were sequentially optimized by selecting the condition that yielded the lowest average false-positive and false-negative percentages. Masks were used to isolate information for the GUV membrane, content, or both regions by excluding the pixels in the window that had a different label value from the one of the tested GUVs in the label maps. Similarly, constructed datasets were used to compare the GUVs quantification carried out by microscopy with IFC.

### 4.5. Imaging Flow Cytometry

IFC measurements were taken on an Amnis ImagestreamX Mk II imaging flow cytometer. The optical configuration used to record fluorescence data is summarized in Supplementary Table S3. GUVs were selected on a brightfield area-side scatter intensity dot plot, excluding the other aggregates, oil inclusions, and internal calibration beads that flow together with the sample [30]. Samples were diluted 1:10 in HS prior to their acquisition. In-focus objects were gated on the brightfield gradient-RMS histogram. From this subpopulation, single objects were selected based on their aspect ratio in the brightfield channel. The gating strategy is summarised in Figure S15. The sample size was set at 10,000 GUV, with an upper limit of total events at 50,000 to limit the file size in dilute samples. IDEAS software 6.2 was used to elaborate the raw image files acquired. A compensation matrix was generated using GUVs stained with isolated fluorophores. Analysis gating followed a similar logic to the one used for acquisition. GUVs were selected based on their area and low side scatter in a brightfield area—side scatter intensity dot plot. Isolated GUVs were then selected based on their aspect ratio. Finally, single vesicles were assigned to populations positive for fluorophore 1, fluorophore 2, or both fluorophores in a fluorophore 1 intensity—fluorophore 2 intensity dot plot in a logarithmic scale, with gates set empirically based on the distributions of the isolated populations. Figure S15 provides further visual information on the gating strategy. A similar logic was applied to the lipid exchange and content exchange experiments.

### 4.6. GUVs Aggregation by Sodium Chloride

GUVs aggregation was induced by incubating 10 µL of 1:1 P1-P2 mixes in 90 µL of sodium chloride solution (250 mM sodium chloride, 50 mM HEPES pH 7.2). The osmolarity of all solutions used was adjusted by measuring it using a Typ3M osmometer (Löser). After 20 h of incubation at room temperature (or 37 °C for MPPC–DOPE 9-1 content exchange), the top 90 µL of solution was removed by aspiration and 90 µL of HS were used to wash the GUVs sediment and induce GUVs separation. After resuspension in an appropriate volume (around 20 µL), the GUVs were imaged as described in Section 4.1.

## 5. Conclusions

We demonstrate a high-throughput microscopy method that permits the quantification of vesicle subpopulations with different fluorescent probes and minimal user input. This method defines vesicle subpopulations based on their membrane and lumen fluorescence properties to identify fusion products and quantify their prevalence in heterogeneous GUVs samples. We highlight the criticalities in sample preparation and compare various analytical approaches to minimize false-negatives and false-positives in the fused population. Our methodology performs on par with state-of-the-art techniques and is readily applicable, since it is based on widely used software including ImageJ and R. Furthermore, our findings can be used and integrated in existing analysis systems to enhance their flexibility in future applications. We applied this fusion quantification method to aggregated GUVs populations in sodium chloride, demonstrating its previously unreported fusogenic activity on a variety of membrane compositions. Fusion events induced during sodium chloride aggregation may be unwanted artefacts in GUVs assembly experiments, or desirable in artificial cell application, where content exchange can be induced in hemifused GUVs clusters.

## Figures and Tables

**Table 1:** Labelling of the GUV populations for lipid and content exchange experiments ^1^.

Name	Lipid Exchange	Content Exchange
P1	DOPE–Af488 0.6%; Dex–CB 40 µM	DPPE–Rh 0.4%; Dex–Af488 40 µM
P2	DOPE–Cy5 0.6%; Dex–CB 40 µM	DPPE–Rh 0.4%; Dex–Af647 10 µM
NC	P1 and P2 mixed 1:1	P1 and P2 mixed 1:1
PC	DOPE–Af488 0.3%; DOPE–Cy5 0.3%; Dex–CB 40 µM	DPPE–Rh 0.4%; Dex–Af488 20 µM; Dex–Af647 5 µM

^1^ For both content and lipid exchange characterization, four GUVs populations were prepared by emulsion transfer, with the fluorophore combinations specified here. Fluorophores are abbreviated as follows: 1,2-dipalmitoyl-sn-glycero-3-phosphoethanolamine-Rhodamine (ammonium salt) as DPPE-Rh; Dextran-Alexa Fluor™ 647 (10,000 MW, anionic, fixable) as Dex–Af647; Dextran-Alexa Fluor™ 488 (3000 MW, anionic) as Dex–Af488; 1,2-dioleoyl-sn-glycero-3-phosphoethanolamine-N-(TopFluor^®^ AF488) (ammonium salt) as DOPE–Af488; 1,2-dioleoyl-sn-glycero-3-phosphoethanolamine-N-(Cyanine 5) as DOPE–Cy5; and Dextran–Cascade Blue™ (10,000 MW, anionic, lysine fixable) as Dex–CB.

## Data Availability

All data generated or analyzed during this study are included in this published article and online materials. The code applied during image data processing is available at https://doi.org/10.5281/zenodo.7782796 (accessed on 29 March 2023). The microscopy dataset and analysis scripts are available at https://doi.org/10.5281/zenodo.7782767 (accessed on 29 March 2023). IFC data with analysis templates can be found at https://doi.org/10.5281/zenodo.7767198 (accessed on 29 March 2023).

## Supplementary Materials

The following supporting information can be downloaded at: https://www.mdpi.com/article/10.3390/ijms24098241/s1.

## Appendix A

### Appendix A.1. Image Processing Details

Object segmentation involves the definition of the background and foreground areas of an image, and is determined by pixel properties (e.g., intensity). Fluorescent areas of giant unilamellar vesicles (GUVs) marked by lipid or content markers must be classified as foreground automatically by applying a filtering sequence to the raw images. To segment the compartments from the micrographs, an ImageJ macro was developed using the ImageJ macro language. The large number of pre-built functions and packages accessible by this language were employed to design an automatic thresholding and labelling system that could analyze fluorescent images of the GUVs without from the need of user input, and regardless of the number of objects contained in each frame. User input is required upon running the script to provide the following information:

- An input folder, where the multi-channel multi-point images are saved. We used Nikon’s nd2 format, although any other format compatible with ImageJ’s Bio-Formats Importer would work as well. A naming convention for the files contained in the input folder was assumed, in the form “DATE_SAMPLENAME_REPLICATE_.nd2”.
- An output folder, where the processed files are going to be saved. Two subfolders are created in the output folder. “ELAB_IMGs” is used to save the contrast adjusted images in a compressed jpg format. Single channels and label maps are saved to inspect the image quality and segmentation outcome in the various files. A merged image of the various single channels is also saved and used for visualization purposes in the later steps of the analysis. “RAW_Data” will contain GUV information in csv format, tiff files for every channel analyzed, and tiff versions of the labelled GUV masks.
- The channels contained in the images, subdivided as content markers or lipid markers, identified by their channel number.
- An overlap percent between the neighboring tiles in the case that this has been set during acquisition. Images are cropped according to their placement in the area scanned. For example, they can be arranged in a snake-by-rows fashion. The overlap issue can be ignored by setting the value to zero, making image indexing unimportant. If a crop percentage above zero is provided, images will be cropped on the appropriate sides by half of the amount indicated, thereby removing overlapping areas from the dataset.

Images in the input folder are opened sequentially in batch mode (by default). Frames are assumed to be arranged in a square. After cropping, contrast adjusted jpgs are saved for all frames. The merged image is generated with up to six channels by automatically assigning lipid markers and content markers to the LUTs in the following order:

- LE: 1—Magenta; 2—Green; and 3—Blue.
- CE: 1—Cyan; 2—Yellow; and 3—Red.

After this, a filtering sequence is applied to each lipid channel to segment the GUVs stained with that specific probe. The following steps are applied in this sequence:

- ImageJ built in the subtract background function is used to apply the rolling ball background subtraction using a radius of 10 pixels.
- Contrast Limited Adaptive Histogram Equalization (CLAHE) [53] from the homonym plugin is used to enhance the contrast between the membranes and the background signal. Block size was set at 99, histogram bins at 256, and maximum slope at 2.0.
- The unsharp mask filter is applied to the contrasted image to enhance contrast. It subtracts a weighted version of the image blurred by a gaussian blur filter, with the radius of the gaussian filter as 1.5 pixels and the weighting factor 0.9.
- The noise in the image is partially suppressed using a gaussian blur filter with standard deviation of 0.5 pixels.

The resulting image therefore has contrast-enhanced and sharpened membrane signals and is used to form a binary mask of membrane regions through automatic thresholding. It is converted to an 8-bit format and thresholded using the Phansalkar’s method [54] with the following settings: radius (5), parameter1 (0.25), and parameter2 (3). The MorphoLibJ plugin was used to obtain masks of GUV lumens [55]. In the first step, membrane regions touching the image border are removed with the “kill borders” function, excluding from the analysis any GUVs that were not completely imaged. Regions bound by membranes were included by a flood fill operation via the “fill holes” function. The difference between the membrane mask without any GUVs on the borders and the filled in mask provides a content-selective mask where the random noise is removed as well. A second clean-up step to remove the artefacts that arose by noise amplification in empty images was applied using the shape filter plugin [56]. The particle area was filtered between 1 and 500 µm^2^, and the solidity was limited between 0.85 and 1. If multiple lipid markers are specified, these operations are performed for each one, and the single channel masks are combined with an OR operation, thereby obtaining a final mask containing information from every lipid channel.

The connected component labelling function included in MorphoLibJ was used to assign a univocal label to each compartment detected using 4-way connectivity. By applying a two-pixel-wide dilation to these labels, an inclusive label map is produced, containing both the GUV lumen and membrane. The difference between the initial mask and the dilated one yields two-pixel-wide rings around the vesicle’s lumen, which are subsequently used as membrane label map. These label maps were used to obtain membrane fluorescence information, using the membrane label map for each membrane channel, or the morphology and lumen fluorescence information using the lumen label map for each content marker. All these values were saved as csv files in the “RAW_Data” folder. Fluorescence information includes the mean pixel intensity, standard deviation, maximum, minimum, median, mode, skewness, and kurtosis. Morphology of the object is saved as perimeter, area, circularity, centroid X and Y coordinate, fitted ellipse X and Y centre coordinates, minor and major axis, ellipse orientation, elongation, convex area, and convexity of the shape (similar to solidity). Values for all the parameters used in the filtering steps were optimised with a version of the macro that accepted the test values for each parameter as vectors (one per parameter) and performed the filtering and detection sequence on a set of test images. The parameter values were tested sequentially, choosing the value that yielded the highest number of detected objects each time. A version of the optimization routine that checks for each combination of values was also implemented, but the run time to test all combinations of parameters was not practical, so it was subsequently used to conduct a small local search, highlighting the minimal effect of the different combinations of parameters.

### Appendix A.2. Statistical Analysis by R

Further data elaboration and statistical treatments are carried out using a template R script that requires input of experimental parameters to summarize the results. Specifically, the following information must be provided in the header of the template to have a complete analysis:

- Working directory (the same as ImageJ output).
- Thickness of the imaging chamber.
- Objective magnification and pixel to micron conversion factor.
- Image pixel width. Images are assumed to have a 1:1 aspect ratio.
- Fraction of non-overlapping pixels as set during acquisition.
- Number of the images per sample, for which a square arrangement is assumed.
- Volume of DS used in the sample preparation.
- List of content markers and lipid markers channels as numeric vectors, indicating the number of the channel in the original image. The elements of these vectors can be named with the type of fluorophore used to correctly label the axes in the plots produced by the analysis routine.
- Path to raw data and elaborated images in a vector containing “RAW_Data\\DATE” and “results.csv” as elements for the raw data folder and “ELAB_IMGs\\DATE” for elaborated images location.
- A vector containing the sample names in the format “SAMPLENAME_REPLICATE” that identifies the files related to a given sample.
- A vector that specifies which samples to use as the controls. The analysis routine expects to find one control for each content marker or lipid marker (depending on if the experiment is content exchange or lipid exchange, respectively), including a negative control where the fluorophores are present in separate populations, and a positive control where the fluorophores are present in all vesicles.
- A vector specifying the areas of the sample droplets in the imaging chamber. For maximum accuracy, these were obtained from the low-magnification map of the whole imaging chamber in ImageJ, by thresholding the edge of the sample droplet, and filling in the whole area with the fill holes function. The areas thus obtained in µm^2^ are multiplied by the spacer width and converted to milliliters.
- A vector containing the z offset used in collecting each sample for size compensation purposes.
- A vector containing the dilution factors used in preparing the samples, to correct the volume of sample calculated by this factor and compare the actual concentrations of the samples.

Data in csv format is for each sample specified are imported and then combined in a single dataset for each element of the samples vector. These datasets were further elaborated by constructing a list containing the datasets and incorporating information from the control samples specified. A background value for each marker was estimated as the average minimum pixel intensity for GUVs of the controls that do not include the marker. This value is subtracted from the mean and maximum fluorescence signal of all datasets. In addition to the adjustments to the fluorescence values, size compensation was also performed. GUVs were modelled as ellipsoids with rotational symmetry around their minor axis. This geometrical assumption was used to compensate the minor and major axis as according to the known axial offset. The compartment volume was calculated from these compensated dimensions, and the radius was taken as the radius of a sphere of an equivalent volume. The shape used here loses validity in deformed GUVs (being of oblate, prolate, stomatocyte or dumbbell shape) but should be a good approximation for spherical or near spherical GUVs. A schematic view of the size adjustment assumptions is presented in Supplementary Figure S11. Finally, correlation data was added for each pairwise combination of channels of the same type (lipid or content markers). For each object in the various datasets, the tiff images of its fluorescence channels were imported. A square region of interest cantered on the GUV (fitted ellipse coordinates) was used to define the region on which the correlation was computed. Pixel values for the two channels considered were converted to numeric vectors and the correlation was computed by the standard correlation test routine in R. This implements the correlation estimates by the Pearson Correlation Coefficient (PCC), Kendall’s coefficient, or the Spearman’s coefficient (the latter two tests are non-parametric). Alternatively, the Manders colocalization coefficient (MCC) was implemented in two variants. The first utilizes the pixel median value as the threshold, while the second uses the minimum pixel value in the GUV area instead. Masks can be used to select areas of the region of interest based on the label value of the GUV of interest. With this method, pixels relative to the GUV membrane, content, or both areas can be isolated from the rest of the region of interest. Thresholding of the fluorescence and correlation histograms was used to define positive GUVs to markers or correlation by using the multimode R package to ultimately find the antimode of the histograms. After thresholding, GUV concentrations and relative population percentages were calculated. These functions were contained in the “GUVfusion_dataElabFunctions.R” script that was imported in the header of the template. Data visualization functions are implemented in “GUVfusion_plotFunctions.R”, allowing the rapid visualization of GUV properties via histograms, dot plots, violin plots, and GUV image composites to visualize the objects classified in the various populations.

### Appendix A.3. Dynamic Light Scattering

Vesicles prepared by the rehydration method at 55 °C were extruded through a polycarbonate membrane with a pore size of 400 nm using an Avanti mini extruder system. After extrusion, the large unilamellar vesicles (LUVs) produced were loaded in a Malvern folded capillary chamber and measured with a Malvern ZetaSizer Pro. A temperature scan from 55 °C to 25 °C was programmed with steps of −0.5 °C, registering the zeta potential at each step to measure its shift with the rearrangement of lipids during phase transition [42].

### Appendix A.4. Simulated Datasets

Three models for content exchange in hemifused pairs of GUVs were constructed. In the first case, full fusion leads to the complete mixing of content markers and dilution based on the sum of the two GUV volumes. Pairs of GUVs from the isolated P1 and P2 datasets were randomly selected. Their fluorescence signals in both content marker channels were summed and scaled by the ratio of the original GUV volume to the sum of the two volumes. The mean intensity in the GUVs imaged through confocal systems was found to be proportional to the fluorophore concentration via the voxel volume, which was not relevant to the dilution factor calculated. In the second model, the hemifused state remains stable over the experiment, but the two GUVs can exchange contents through the diaphragm via pore formation and resealing. The fluorescent signals of the two randomly selected GUVs were exchanged based on a random number generated uniformly in the interval [0; 1). The GUV transferred a proportion of the fluorescence signal to the other one proportional to the random number, while it retains the remaining proportion (one minus the random number). No dilution was considered in this model. The third model is a variant of the second one, where the likelihood of exchange of one fluorophore is larger than the other. For example, if one fluorophore has a larger diffusion coefficient or membrane permeability, its exchange based on its diffusive properties will be predominant compared to the other fluorophore. Two random numbers were generated, which dictated the exchange of fluorophore one and fluorophore two. The first was in the range [0.5; 1), while the second was in [0; 0.5). Results of these simulated fluorescence exchanges were plotted in a fluorescence dot plot in red, with the original points from the negative control dataset in gray as a reference. The results are presented in Supplementary Figure S16.
