# Supplementary material for: Quantification of Giant Unilamellar Vesicle Fusion Products by High-Throughput Image Analysis
Source: Int J Mol Sci. 2023 May 4;24(9):8241. doi: 10.3390/ijms24098241 (PMC10179211; doi:10.3390/ijms24098241)
Supplement: Supplementary file 1 [file ijms-24-08241-s001.zip › ijms-2345597-supplementary.pdf]

Figure S1

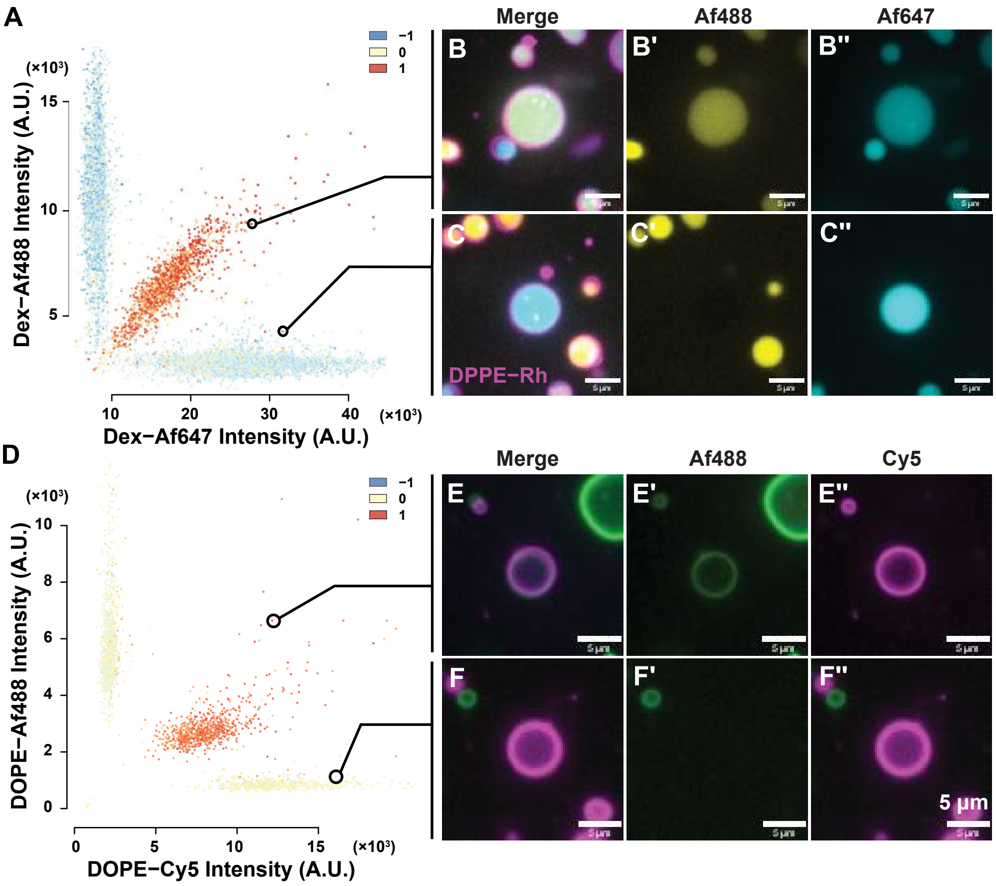

Figure S1 Classification of positive GUV based on fluorescence correlation. Fluorescence dot plots with point colour representing correlation score for content exchange (**A**) or lipid exchange (**D**) mixed samples. GUV with both content markers (**B**, **B'**, **B''**) or lipid markers (**E**, **E'**, **E'**) are easily visible by their higher correlation scores compared to GUV with only one marker (**C**, **C'**, **C''** for content and **F**, **F'**, **F''** for lipid markers).

Figure S2

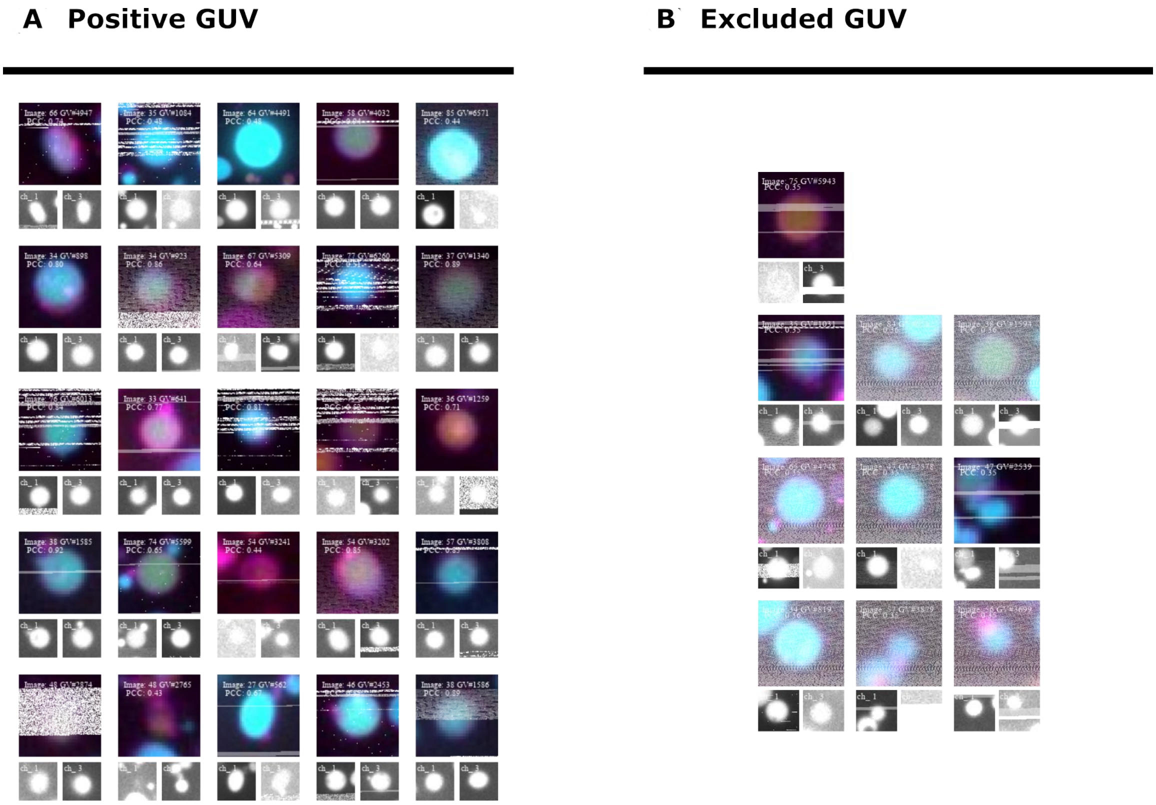

Figure S2 Visualization of positive GUV.

Panels composed of GUV images with single channel representation and merged image to show the presence of fluorophores in selected populations. (**A**) shows a composition of GUV selected from the content exchange-positive population. (**B**) shows a panel of GUV excluded from the positive set and considered false positive due to their low colocalization score. The images show GUV from the content exchange experiment described in Section 2.4. Vesicles from this experiment contained either Dex−Af647 (left inset images) or Dex−Af488 (right inset images) or both fluorophores. Some images present noise and artefacts produced during the panel composition and not visible in the original images.

Figure S3

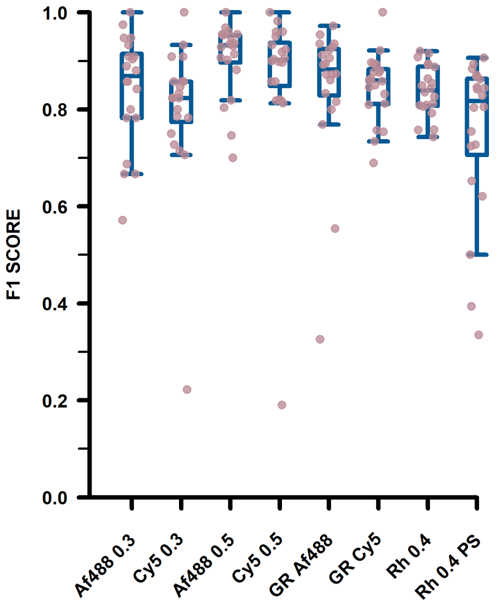

Figure S3 GUV detection quality by F1 score.
Analysis of GUV detection reliability based on the F1 score for the same samples presented in Figure 2. GUV produced by gentle rehydration (GR Af488, GR Cy5) and emulsion transfer (Af488 0.3, Af488 0.6, Cy5 0.3, Cy5 0.6, Rh 0.4, Rh 0.4 PS) show similar average F1 scores. The presence of multilamellar vesicles, phase separated membranes or lower fluorophore concentrations lead to lower F1 scores.

Figure S4

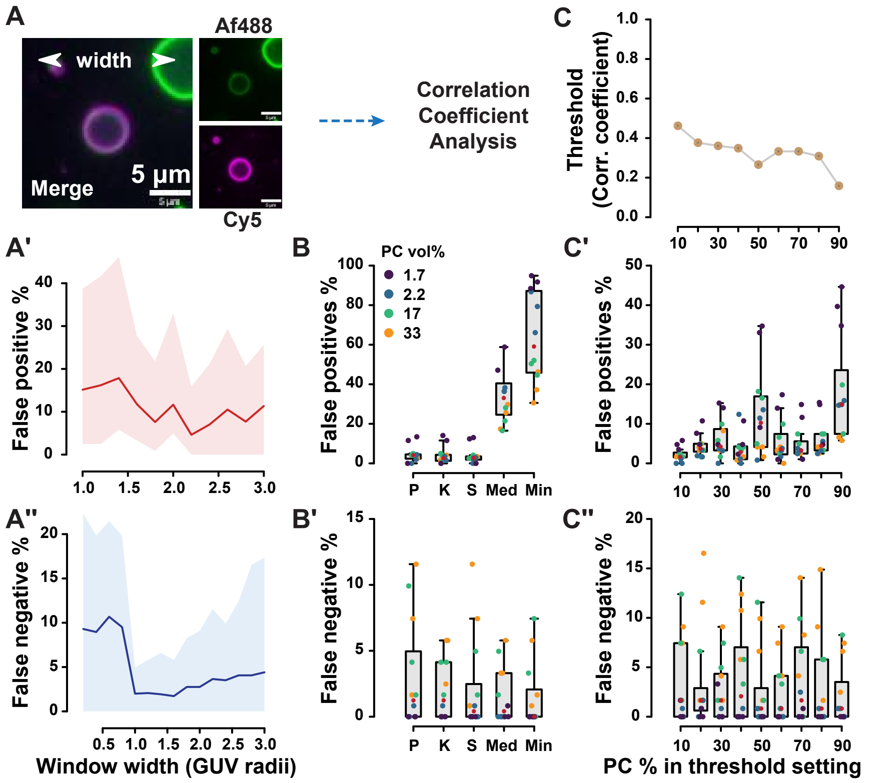

Figure S4 Optimization of parameters for lipid marker correlation.

Optimization of parameters involved in correlation evaluation in lipid exchange controls. The first parameter considerd for correlation is the size of the region of interest around each vesicle, expressed as a fraction of its radius. Window width (**A**) showed a dramatic decrease of both false positive (**A'**) and false negative (**A"**) percentage above one GUV radii, highlighting the importance of including the entire GUV in the window. The optimum chosen was 2.2 GUV radii. After width optimization, a variety of correlation metrics were compared (Pearson, Spearman, Kendall, Manders with median and minimum thresholding). The lowest error rates were found for non-parametric Spearman and Kendall coefficients (**B**, **B'**). Pearson correlation coefficient had similar error rates while Manders colocalization coefficient displayed frequent misclassification (maybe due to non-optimal thresholding strategies). Labels in **B** and **B'** stand for Pearson (P), Kendall (K), Spearman (S), Manders with median thresholding (Med) and minimum thresholding (Min). The last paramter tested was the fraction of positive control vesicles used in threshold setting on a dataset formed by random sampling of positive and negative vesicles from controls. Threshold setting showed a small dependence on the percentage of positive control vesicles used (**C**). This had low impact on false negative rates and tended to make false positives more frequent as thresholds get lower (**C'** and **C"**). The fraction of positive GUV was set at 0.4. Masks to select image areas for analysis were tested but did not yield appreciable separation between positive control and negative control.

Figure S5

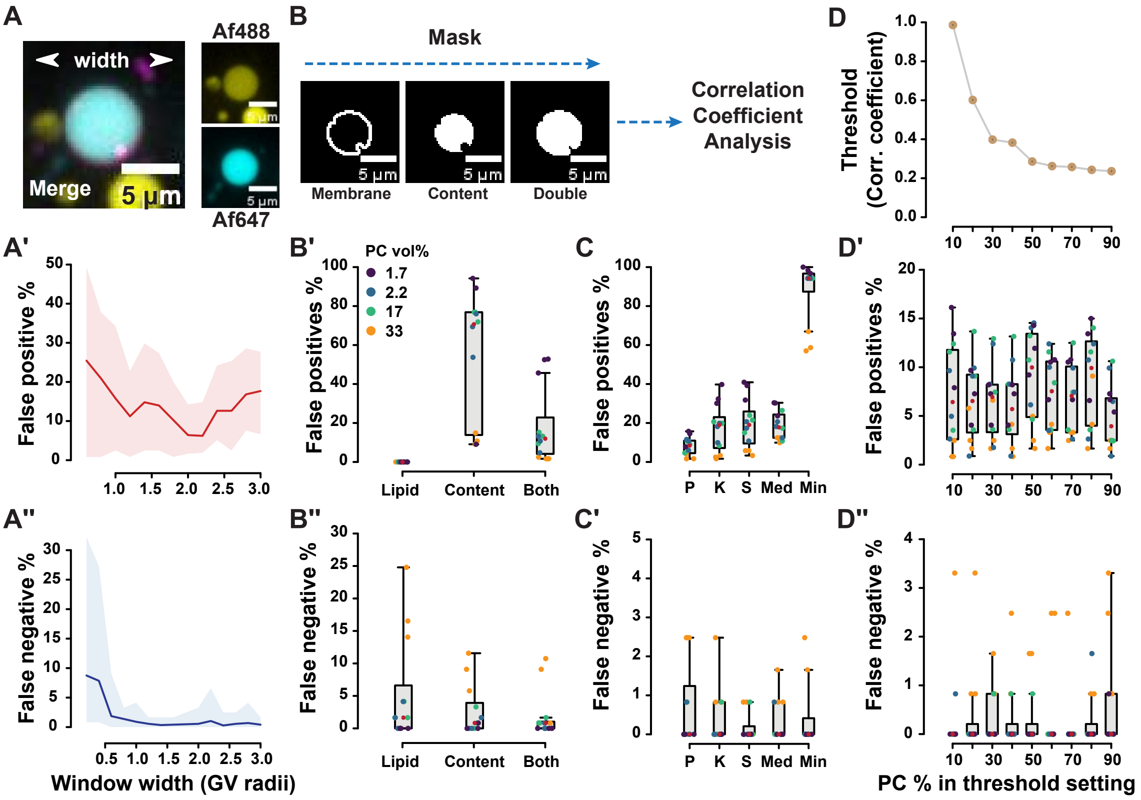

Figure S5 Optimization of parameters for content marker correlation.

Optimization of parameters involved in correlation evaluation in content exchange controls. The sequence of optimization step is the same used in Figure S4. Window width showed a similar trend to that found for lipid exchange, and it was set at 2.2 (**A**, **A'**, **A''**). In this case, mask use to select pixels related to a single GUV allowed differentiation of positive and negative vesicles. Mask use to select relevant pixels from the image lead to more false positive errors, so no masking approach was used in further analysis (**B**, **B'**, **B''**). The lipid mask selected too few pixels, making correlation not significant in most objects, the 0% false positive rate is an artefact due to the lack of GUV in the positive category. Among the correlation coefficient tested, PCC performed the best (**C**, **C'**). Labels in **C** and **C'** stand for Pearson (P), Kendall (K), Spearman (S), Mander's with median thresholding (Med) and minimum thresholding (Min). Threhsold setting behaved as described for lipid exchange, with low dependency of error rates to threshold setting parameters. 0.4 was chosen in this case as well (**D**, **D'**, **D''**).

Figure S6

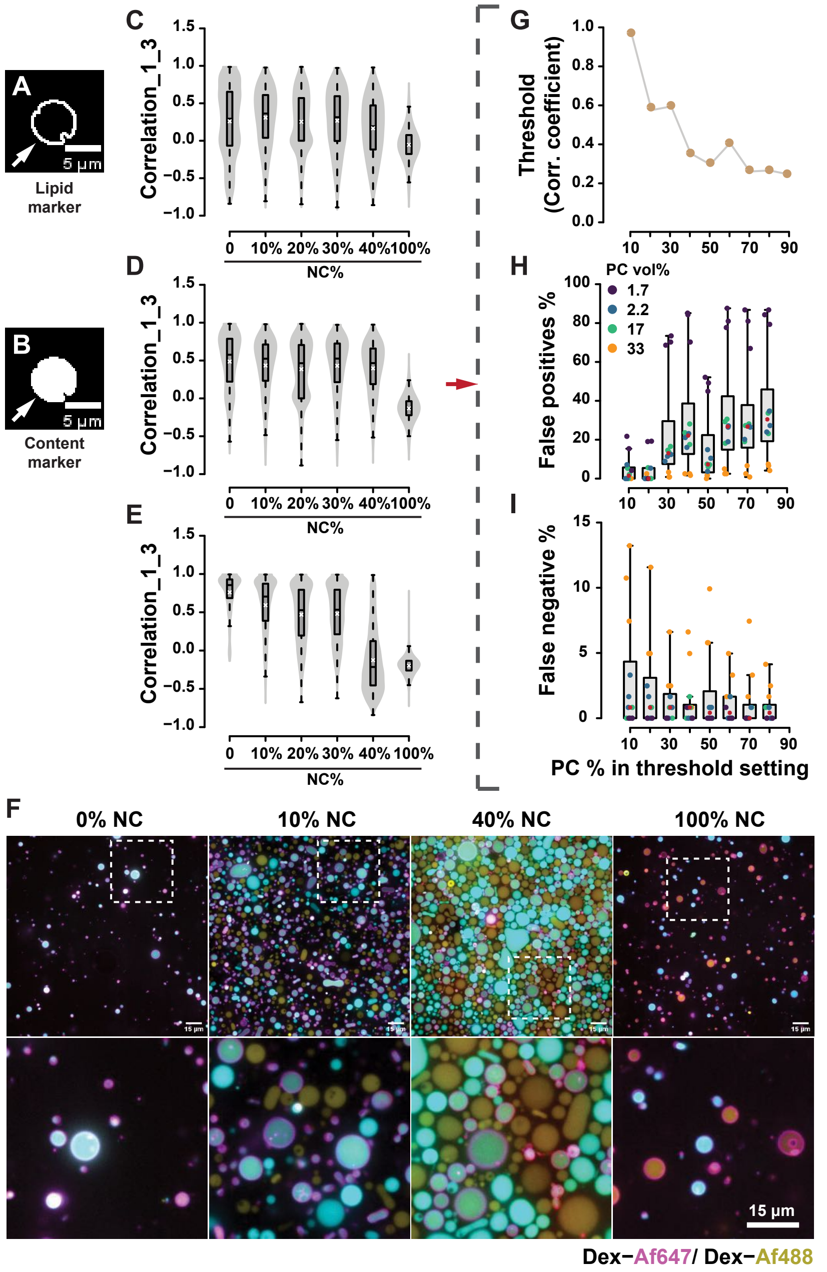

Figure S6 Impact of GUV neighbourhood on correlation score.

Effects of noise and masking on correlation of positive control GUV. Lipid mask (**A**) and GUV mask (**B**) can be used to isolate a GUV fluorescence from the surrounding noise. This also reduces the number of pixels considered and the overall signal variability, making the difference between positive and negative control smaller. **C**, **D**, **E** show the shift in correlation distribution for positive control GUV with increasing amounts of negative control GUV (noise) with lipid mask, content mask and no mask applied respectively. While in **E** the distribution shifts dramatically with increasing noise percentage, the use of a mask limits this effect. The lipid mask uses too few pixels, leading to very little difference between positive and negative control distributions. Since the GUV mask led to improvements in detection with high noise, the thresholding step was optimized for this condition as well, showing increased dependency of the threshold and error rates on the thresholding parameter (**G**, **H**, **I**). This was expected since in this case the negative and positive control distributions overlap significantly. (**F**) shows samples of micrographs taken from the samples with 0%, 10%, 40% vol/vol negative GUVs added and a sample of negative GUV, highlighting the changes in local fluorescence distribution around positive GUV. The 100% NC sample was diluted for visualization purposes.

Figure S7

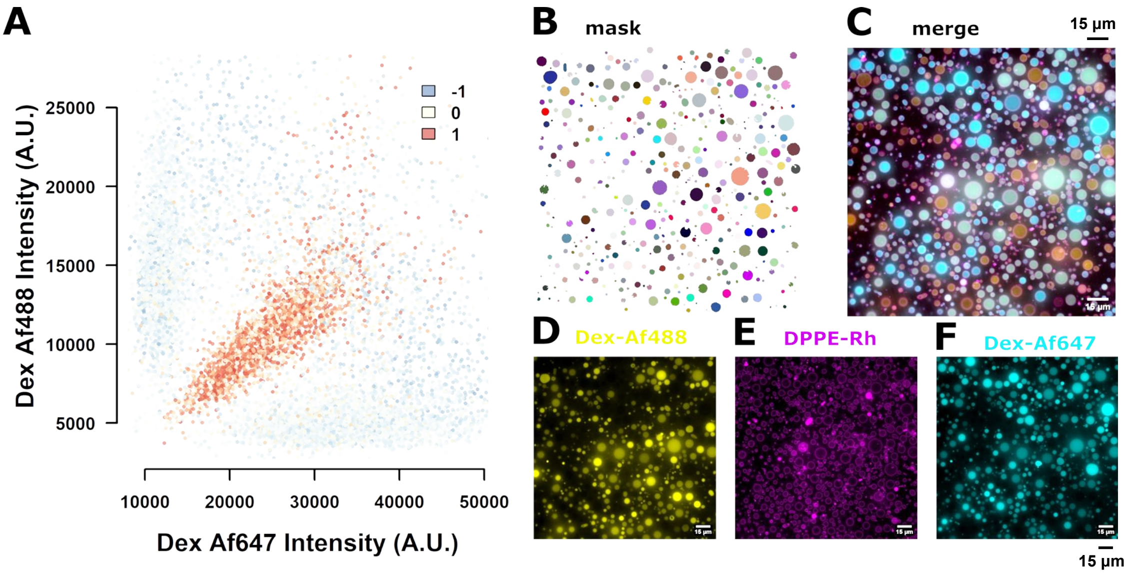

Figure S7 Robustness of correlation metrics to GUV surface density.
Fluorescence dot plot with colour-coded correlation information for a mixed sample containing P1 (Dex−Af488), P2 (Dex−Af647) and PC (Dex−Af488 with Dex−Af647) 1:1:1 in the content exchange experiment (**A**). The sample presented high cross-talk due to diffuse fluorescence and high GUV concentration, highlighted by low-correlation GUV with high signals in both channels. Micrographs of the detection result (**B**), merged channels (**C**) and single channels (**D**, Dex−Af488, **E**, DPPE−Rh and **F**, Dex−Af647) are included as visual indication. Scale bar = 15 µm.

Figure S8

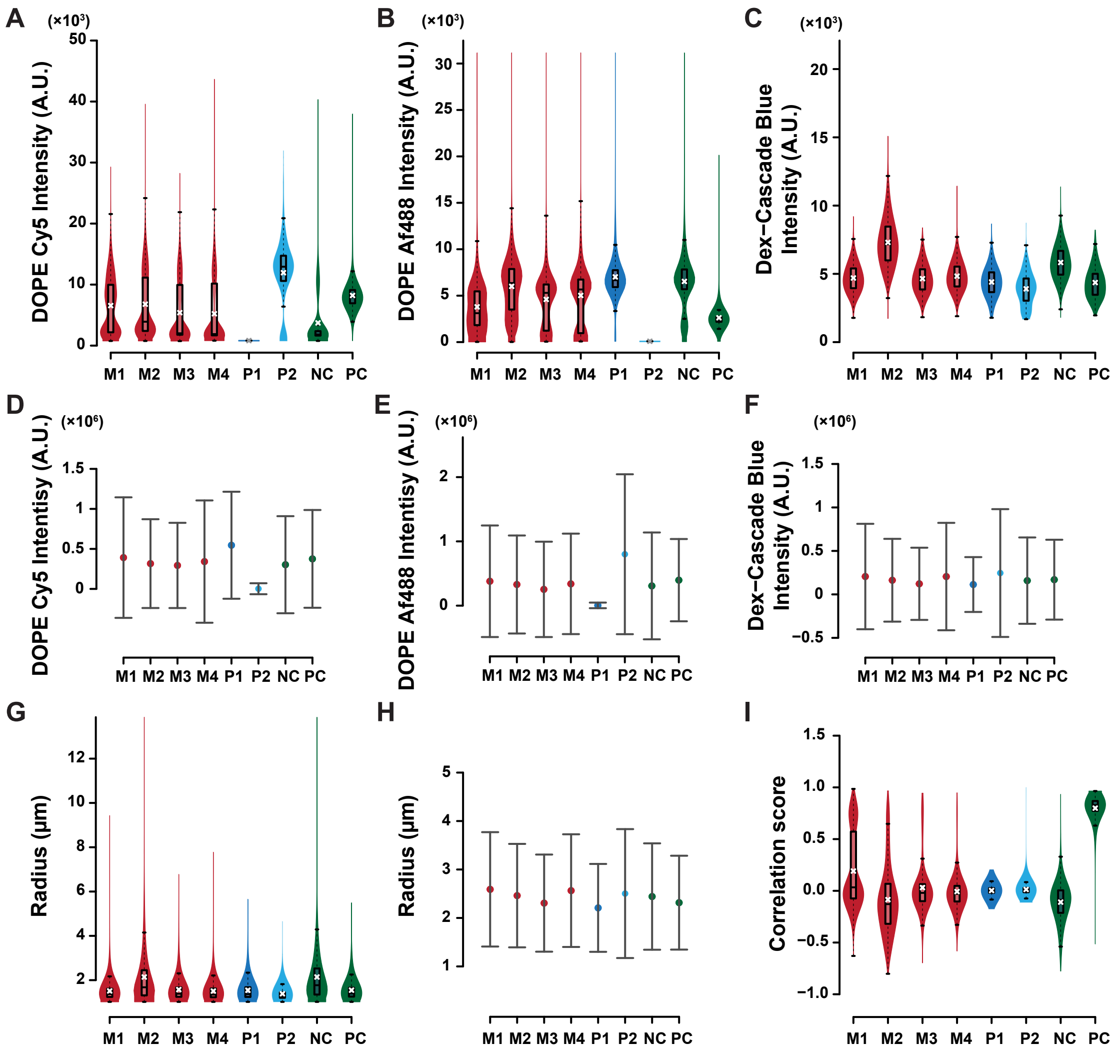

Figure S8 Fluorescence and size distribution summary for lipid exchange control experiments. Comparison of GUV properties between IFC and microscopy for lipid exchange. The figure uses the same dataset used in Figures S4, S5, 4 and 5. DOPE−Cy5, DOPE−Af488, Dex−CB mean fluorescence for microscopy are represented in (**A**, **B**, **C**), while average values for IFC are plotted in (**D**, **E**, **F**) with whiskers representing the standard deviation. GUV radius in microscopy (**G**) is significantly smaller compared to estimate from IFC (**H**). In (**I**) the correlation violin plots are reported.

Figure S9

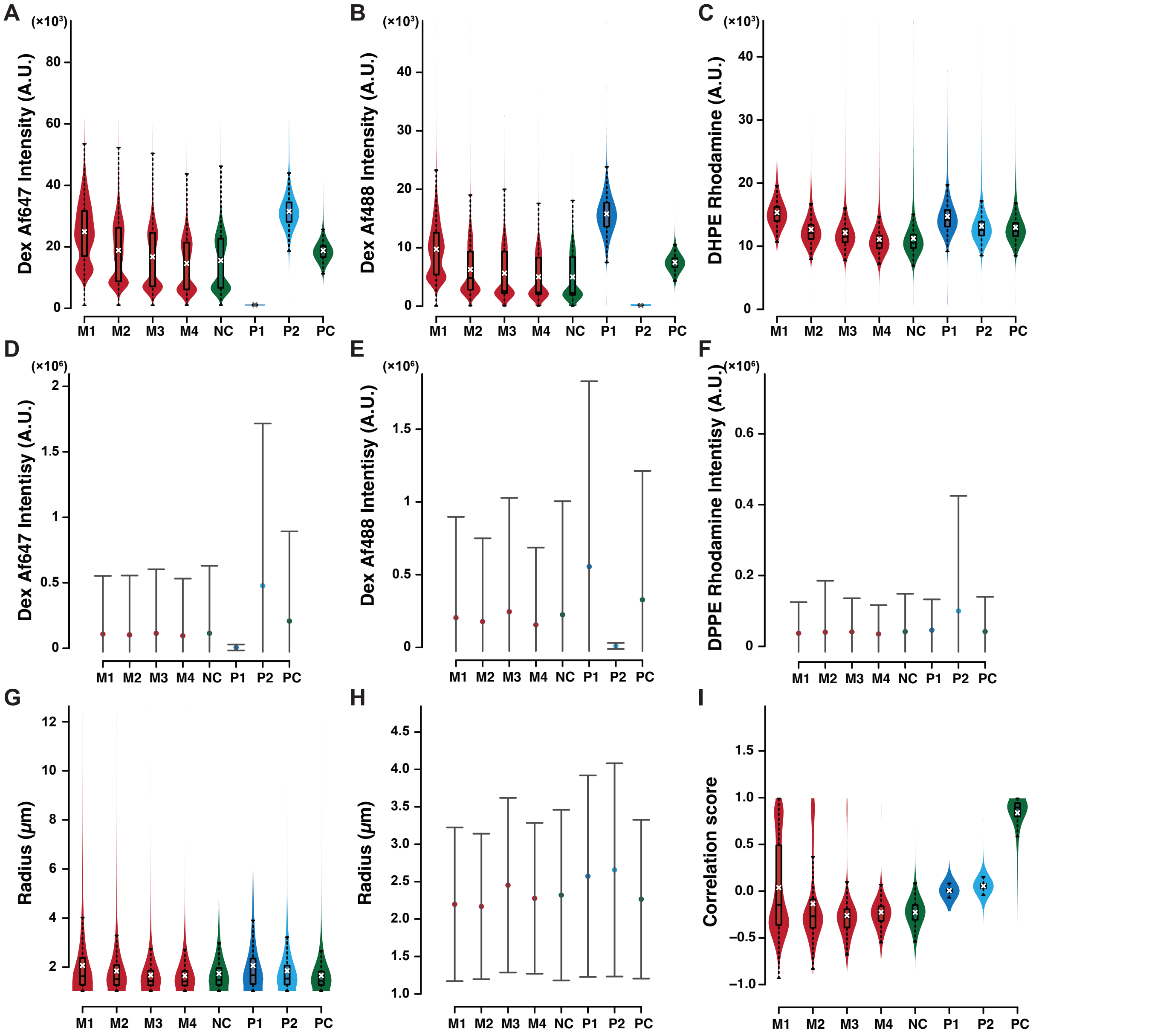

Figure S9 Fluorescence and size distribution summary for content exchange control experiments. Comparison of GUV properties between IFC and microscopy for content exchange. The data comes from datasets used in Figures S4, S5, 4 and 5. Dex−Af647, Dex−Af488 and DPPE−Rh average fluorescence distribution for microscopy (**A**, **B**, **C**) and IFC (**D**, **E**, **F**). Like in the previous case, radius estimates for microscopy (**G**) are lower than those from IFC (**H**). (**I**) shows correlation distributions.

Figure S10

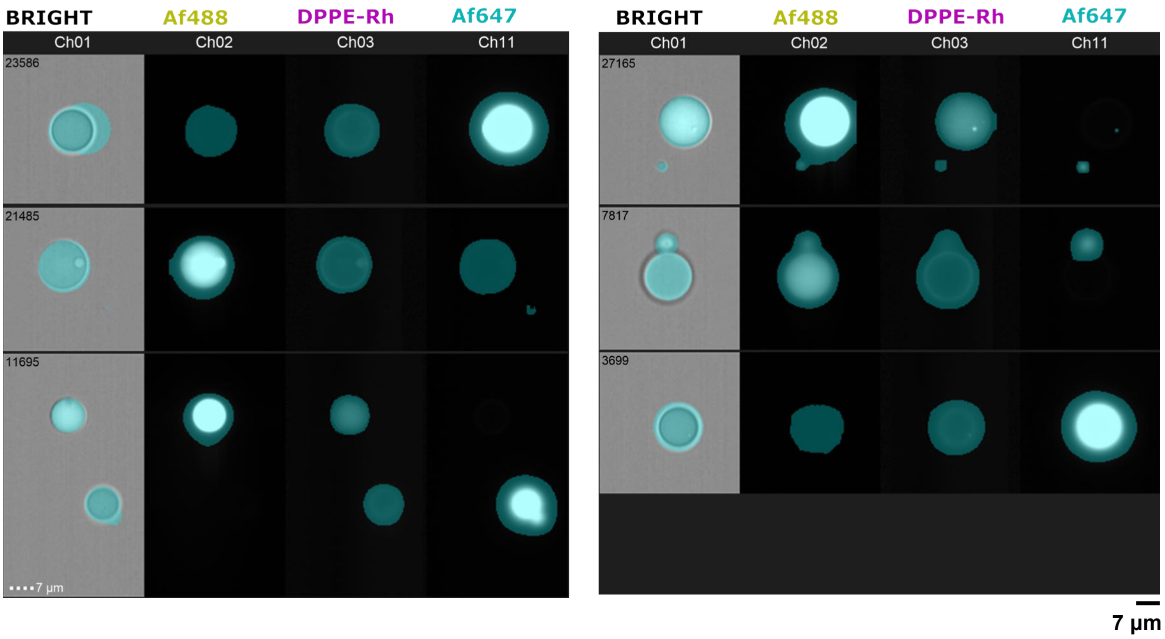

Figure S10 Masks for IFC size estimate.
Samples of GUV acquired from IFC with masks visualized in cyan. The masks used in channel 1 (brightfield) are used for radius estimate and can occasionally overestimate GUV size. Scale bar = 7 µm.

Figure S11

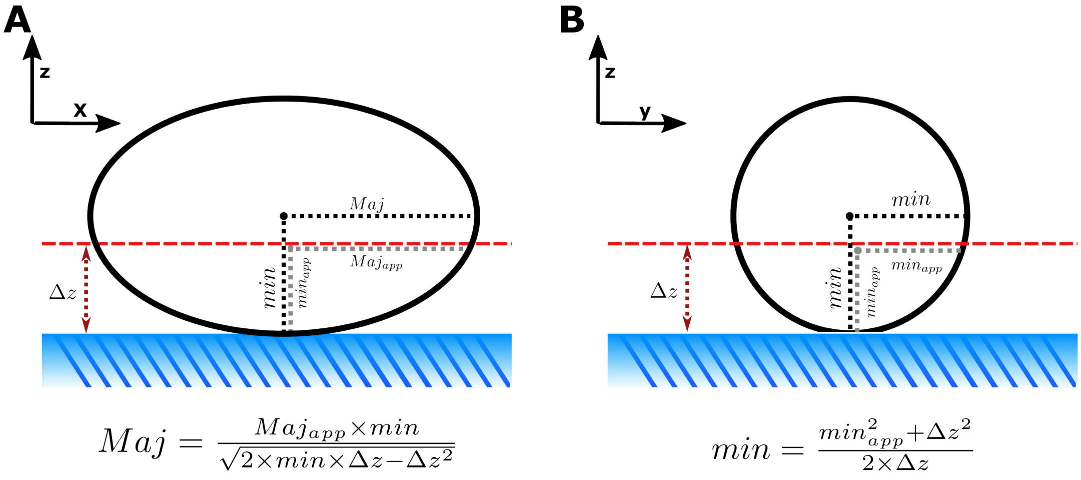

Figure S11 Size compensation.
Scheme of size compensation, showing the intersection of the imaging plane with an ellipsoidal GUV (**A**, **B**) sedimented on a surface.

Figure S12

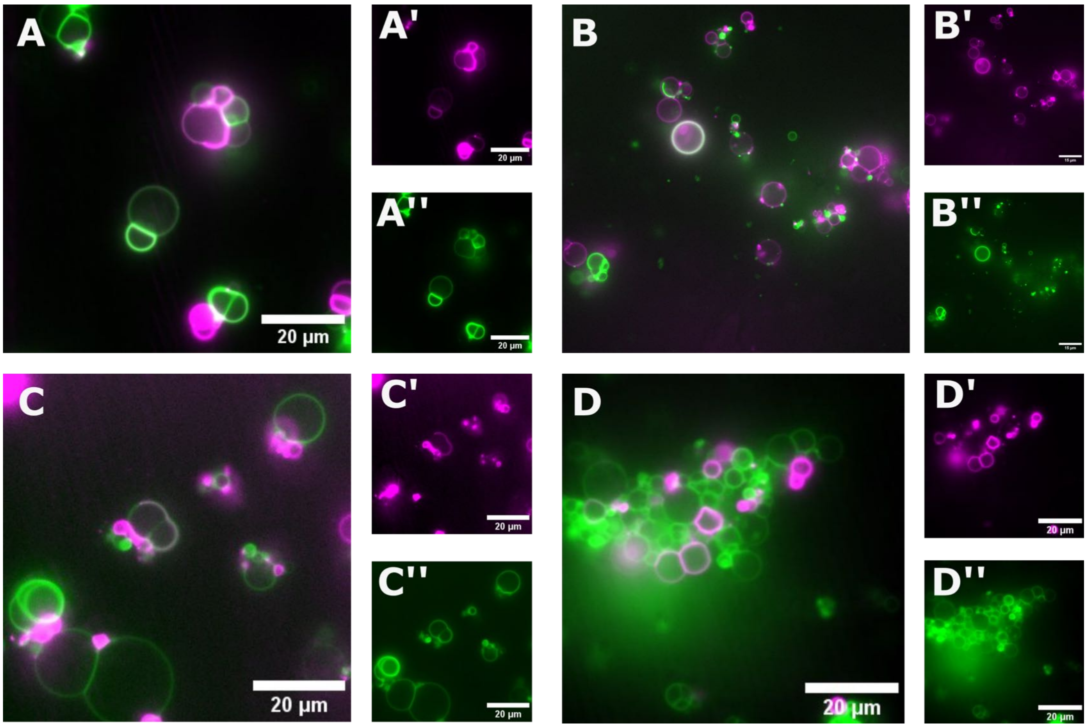

Figure S12 Lipid exchange in gentle rehydration GUV.
Lipid exchange upon sodium chloride aggregation in GUV produced by gentle rehydration made of POPC (**A** merge, **A’** DOPE−Cy5 fluorescence, **A”** DOPE−Af488 fluorescence); 1,2 tridecanoyl-sn-glycero-3-phosphocholine (13PC) - 1,2 diphytanoyl-sn-glycero-3-phosphocholine 9-1 (**B** merge, **B’** DOPE−Cy5 fluorescence, **B”** DOPE−Af488 fluorescence), 13PC-DOPE 9-1 (**C** merge, **C’** DOPE−Cy5 fluorescence, **C”** DOPE−Af488 fluorescence), and 13PC (**D** merge, **D’** DOPE−Cy5 fluorescence, **D”** DOPE−Af488 fluorescence). Two separate populations were stained with 0.4% DOPE−Af488 or 0.4% DOPE−Cy5. Images were taken after 4 hours of incubation in 200 mM Sodium Chloride, 50 mM HEPES, isotonic to the internal solution.

Figure S13

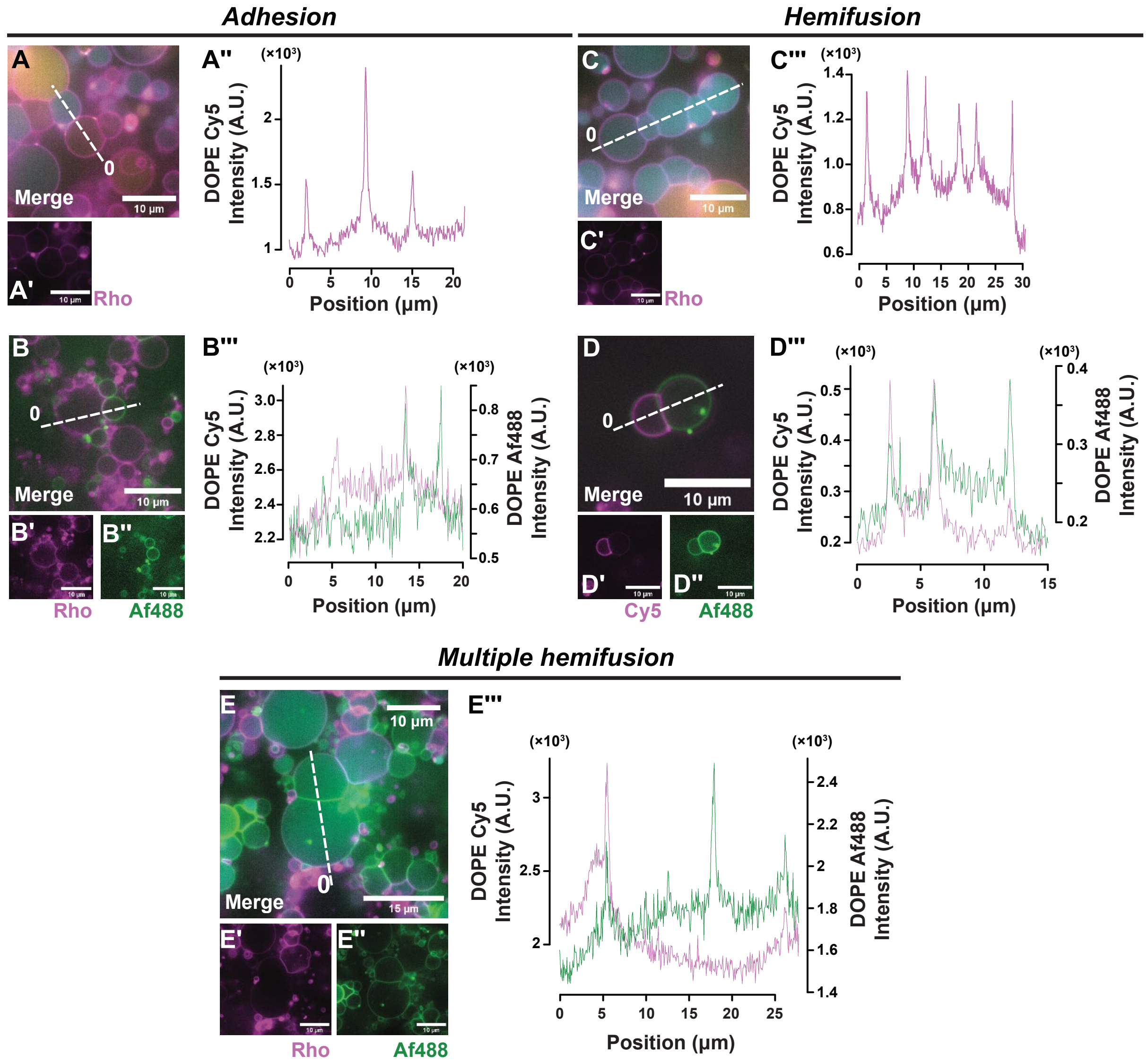

Figure S13 Redistribution of fluorescent lipids with GUV aggregation and hemifusion. GUV interactions change the distribution of membrane probes between interacting compartments. If one marker is included, adhesion (**A**, **A'**) is visible by a peak in the intensity profile of the GUV dimer due to the presence of two bilayers (**A"**) (MPPC-DOPE 9-1 GUV). The same phenomenon in a two-marker experiment (**B**, **B'**, **B"**) is represented by the colocalization of fluorescence peaks at the point of contact (**B'"**) (DPPC-DOPE 9-1). Hemifusion in a single-marker experiment in (**C**, **C'**) is highlighted by the constant intensity of interfaces between associated compartments (**C"**) (MPPC-DOPE 9-1 GUV). In a two-marker experiment (**D**, **D'**, **D"**), lipid transfer of fluorescent lipids from a source (higher fluorescence) to the associated vesicle (DPPC-DOPE 9-1 GUV) is visible. These lipids can only diffuse to the outer monolayer (without flip flop between monolayers) so the intensity on the receiver vesicle equilibrates to a lower value than the donor vesicle (**D'"**). In the case of multiple hemifusion events in a two-marker experiment (**E**, **E'**, **E"**), the diffusion of a second lipid marker to the outer monolayer of the hemifusion state can be observed, with lipids excluded from the interface between the compartments (**E'"**) (MPPC-DOPE 9-1 GUV). Scale bars = 10 µm.

Figure S14

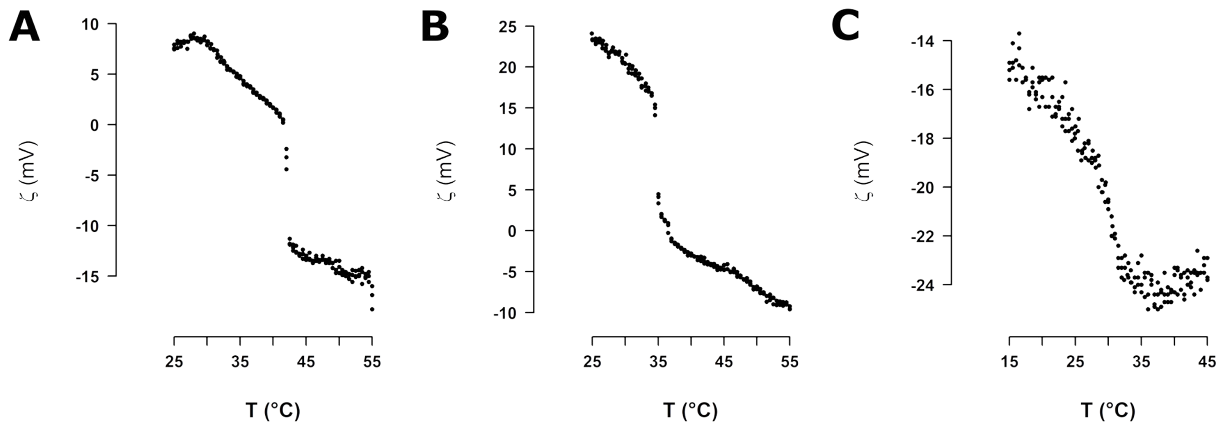

Figure S14 Temperature-dependent zeta potential measurements of various lipid-derived vesicles. DPPC vesicles (**A**), MPPC vesicles (**B**), and MPPC-DOPE 9-1 vesicles (**C**). The transition of the lipids to the liquid ordered state is marked by a sharp change in zeta potential. In the presence of secondary components, the transition is widened when the two lipids phase separate.

Figure S15

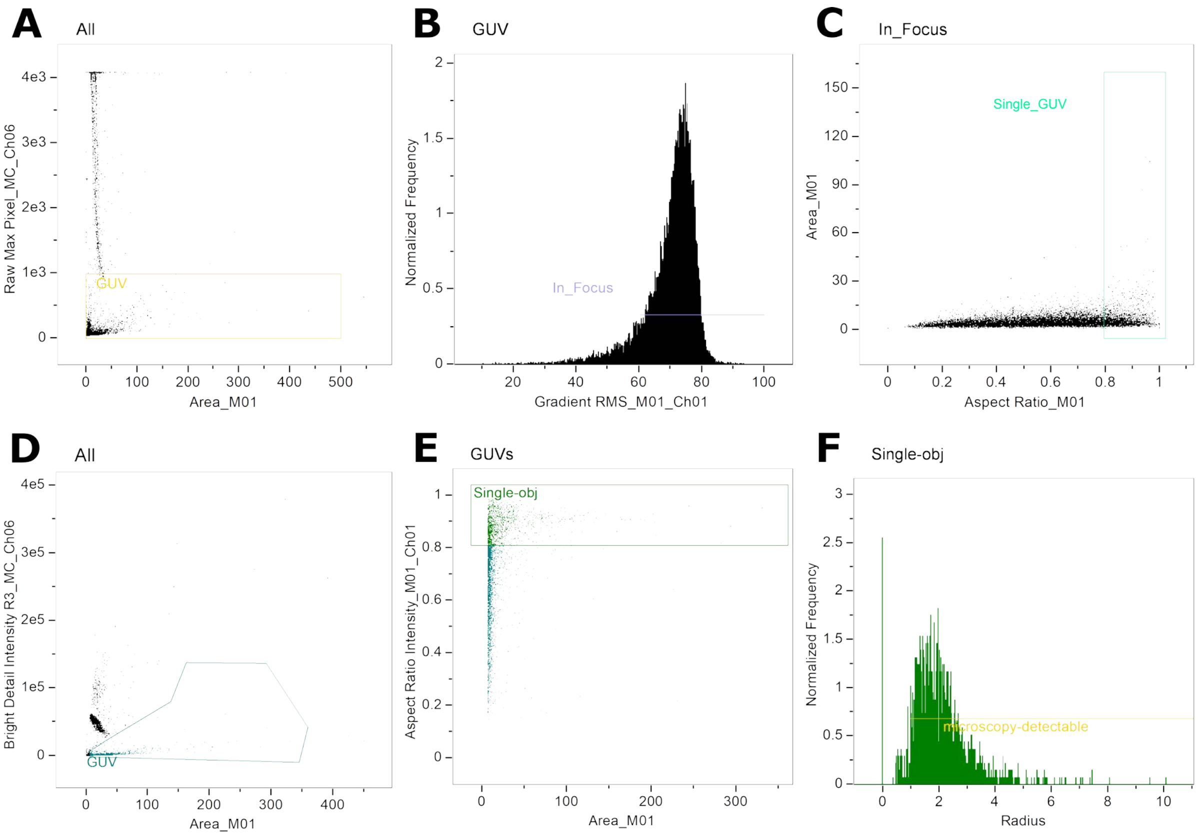

Figure S15 Gating in IFC analysis.
Gating strategy used for IFC acquisition (**A**, **B**, **C**) and analysis (**D**, **E**, **F**, **G**).

Figure S16

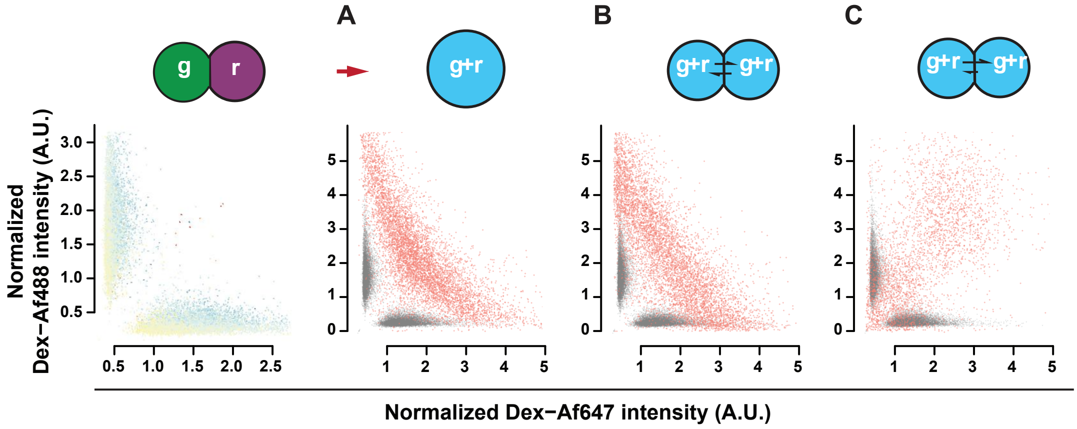

Figure S16 Fluorescence redistribution according to three models of content exchange. Fluorescence dot plots in a negative control sample (grey) and randomly generated mixed GUV according to three models for content exchange: full fusion (**A**); random symmetric content exchange (**B**); preferential exchange of one probe (**C**). (**B**) reproduces well the exchange by sodium chloride incubation observed.

Table S1 Sample sizes summary. Sample sizes for datasets used in IFC and microscopy comparison

	IFC content exchange	IFC lipid exchange	Microscopy content exchange	Microscopy lipid exchange
MIX1_1	1371	3524	17704	2636
MIX1_2	1421	3386	13674	7513
MIX1_3	1556	3846	15696	6891
MIX2_1	1281	3168	8980	7570
MIX2_2	1487	3739	6777	5731
MIX2_3	1561	3359	7034	2581
MIX3_1	1565	2035	6981	1774
MIX3_2	1503	2596	4307	801
MIX3_3	1673	3540	4445	846
MIX4_1	1466	2422	5503	2639
MIX4_2	1479	4171	4544	338
MIX4_3	1469	2605	4643	4233
P1_1	1680	2255	9639	1452
P1_2	1656	2140	8258	1246
P1_3	1613	3941	11724	1679
P2_1	1633	3585	14604	267
P2_2	1611	2190	13682	430
P2_3	1607	2618	10563	477
NC_1	1432	2441	8214	7474
NC_2	1528	2510	7794	5789
NC_3	1510	2447	7161	1676
PC_1	1513	2468	11840	749
PC_2	1543	3419	14201	583
PC_3	1455	3139	9401	215

Table S2 Microscope acquisition specifications. Optical configuration of microscopy acquisitions

Channel	Dichroic (nm)	Emission filter (nm)	Fluorophore imaged
DAPI	425	460/50	Dex−CB
FITC	495	525/50	DOPE−Af488, Dex−Af488
Cy3	565	605/70	DPPE−Rho
Cy5	660	700/75	DOPE−Cy5, Dex−Af647

Table S3 IFC acquisition specifications. Optical setup for fluorescence detection in imaging flow cytometry

Channel	Laser	Bandpass emission	Camera	Fluorophore
2	488 nm	528/65	1	DOPE−Af488;
3	488 nm	577/35	1	Dex−Af488 DPPE−Rh
7	405 nm	457/45	2	Dex−CB
11	642 nm	702/85	2	DOPE−Cy5; Dex−Af647
